# Transcriptomic Comparison of Human Peripartum and Dilated Cardiomyopathy Identifies Differences in Key Disease Pathways

**DOI:** 10.3390/jcdd10050188

**Published:** 2023-04-23

**Authors:** Jude Taylor, Anna C. Y. Yeung, Anthony Ashton, Alen Faiz, Victor Guryev, Bernard Fang, Sean Lal, Mark Grosser, Cristobal G. dos Remedios, Filip Braet, Craig S. McLachlan, Amy Li

**Affiliations:** 1 Centre for Healthy Futures, Torrens University Australia, Surrey Hills, NSW 2010, Australia; 2 Respiratory Bioinformatics and Molecular Biology, School of Life Sciences, The University of Technology Sydney (UTS), Sydney, NSW 2007, Australia; 3 Division of Cardiovascular Medicine, Lankenau Institute for Medical Research, Wynnewood, PA 19096, USA; 4 Groningen Research Institute for Asthma and COPD (GRIAC), The University of Groningen, 9700 Groningen, The Netherlands; 5 Laboratory of Genome Structure and Ageing, European Research Institute for the Biology of Ageing (ERIBA), University Medical Centre Groningen, The University of Groningen, 9713 Groningen, The Netherlands; 6 Charles Perkins Centre, The University of Sydney, Sydney, NSW 2006, Australia; 7 School of Medical Sciences, The University of Sydney, Sydney, NSW 2006, Australia; 8 Department of Cardiology, The Royal Prince Alfred Hospital, Sydney, NSW 2050, Australia; 9 Sydney Heart Bank, The University of Sydney, Sydney, NSW 2006, Australia; 10 23Strands, Pyrmont, NSW 2009, Australia; 11 Victor Chang Cardiac Research Institute, Darlinghurst, NSW 2010, Australia; 12 Australian Centre for Microscopy & Microanalysis, The University of Sydney, Sydney, NSW 2006, Australia; 13 Department of Pharmacy & Biomedical Sciences, La Trobe University, Bendigo, VIC 3550, Australia

**Keywords:** peripartum cardiomyopathy, dilated cardiomyopathy, RNAseq gene expression

## Abstract

Peripartum cardiomyopathy (PPCM) is a rare form of acute onset heart failure that presents in otherwise healthy pregnant women around the time of delivery. While most of these women respond to early intervention, about 20% progress to end-stage heart failure that symptomatically resembles dilated cardiomyopathy (DCM). In this study, we examined two independent RNAseq datasets from the left ventricle of end-stage PPCM patients and compared gene expression profiles to female DCM and non-failing donors. Differential gene expression, enrichment analysis and cellular deconvolution were performed to identify key processes in disease pathology. PPCM and DCM display similar enrichment in metabolic pathways and extracellular matrix remodeling suggesting these are similar processes across end-stage systolic heart failure. Genes involved in golgi vesicles biogenesis and budding were enriched in PPCM left ventricles compared to healthy donors but were not found in DCM. Furthermore, changes in immune cell populations are evident in PPCM but to a lesser extent compared to DCM, where the latter is associated with pronounced pro-inflammatory and cytotoxic T cell activity. This study reveals several pathways that are common to end-stage heart failure but also identifies potential targets of disease that may be unique to PPCM and DCM.

## 1. Introduction

Peripartum cardiomyopathy (PPCM) is an acute form of idiopathic heart failure that manifests towards the end of pregnancy or in the months following delivery [1]. PPCM is a diagnosis of exclusion, but it is complicated by a symptomatic presentation that closely resembles late-stage pregnancy, such as dyspnea and oedema, or dilated cardiomyopathy (DCM)—a common form of systolic heart muscle failure. Several lines of evidence presented suggest the potential involvement of fetal micro-chimerism, myocarditis, genetic mutations, and vasculo-hormonal pathways [2,3]. There remains no clear explanation as to why severe heart failure in the form of PPCM develops in a small but significant fraction of women during late pregnancy. 

PPCM and DCM share several genetic and clinical features. Both populations are genetically predisposed to mutations in shared sarcomeric and cytoplasmic genes [4,5,6]. Clinically, both present symptomatically with a combination of dyspnea, fatigue, ventricular (usually LV) dilatation, systolic dysfunction with reduced ejection fraction (EF < 45%), bi-atrial enlargement, mitral and tricuspid regurgitation, pulmonary hypertension, tachycardia, and peripheral edema [7,8]. Despite these similarities, PPCM and DCM are not identical. PPCM typically presents in previously healthy adults who, in their twenties or thirties, rapidly deteriorate into heart failure. Nevertheless, the condition can be stabilized and recovery of heart function can be expected in nearly half of those diagnosed at the onset of disease [9]. In contrast, DCM is characterized by late onset and slow progression that may be stabilized but is rarely reversed with treatment [10]. Thus, while PPCM is considered a subset of DCM and clinically managed in a similar manner, we suggest there are significant differences. 

In recent investigations, circulating natural killer (NK) and T cells in PPCM patients were identified [11,12]. Similar changes in NK cells and additional T cell subsets were also found in recent onset cardiomyopathy from the same study. Comparatively, DCM exhibited aberrant macrophage, CD4^+^ T and B activity involved in disease pathogenesis and progression in the human heart [13,14,15,16]. Mouse models of DCM, B, and CD4^+^ T cells play a pivotal role in generating cardiomyocyte-specific IgG autoantibodies resulting in autoimmune myocarditis, which is intricately involved in myocardial remodeling [16,17,18]. These observations support the hypothesis that an immune-mediated cellular mechanism may be involved in both PPCM and DCM pathophysiology, although PPCM differs from DCM. To test this hypothesis, we undertook differential gene expression and immune cell deconvolution analysis of LV myocardial tissue from end-stage PPCM. We performed gene set enrichment analysis and cell-type annotations to identify molecular processes and pathways involved in PPCM. These findings were compared to end-stage DCM patients to identify immune cell subsets and pathways that may reflect divergent pathological processes. 

## 2. Materials and Methods

### 2.1. Data Acquisition and Sample Population

Datasets containing human non-failing donors, DCM and/or PPCM from left ventricular (LV) human samples, were downloaded from the Gene Expression Omnibus (GEO) database. GEO human heart datasets used in this study include bulk RNAseq, GSE141910, GSE71613, and GSE55296, as well as single cell RNAseq GSE183852. A second RNAseq dataset of PPCM and donor was acquired from the Sydney Heart Bank database upon request, using the sequencing protocol briefly outlined below. The acquired datasets were imported into R software version 3.4 for subsequent analysis using R packages and Bioconductor. 

PPCM and donor heart tissue was collected as a part of the Sydney Heart Bank Biorepository. PPCM hearts were collected from patients undergoing heart transplantation at St. Vincent’s Hospital Sydney. Donors with non-cardiac causes of death were collected by the Red Cross Blood Services. Human heart tissue used in this study was collected with the informed and written consent of transplant patients or from the families of organ donors of non-failing hearts in accordance with the principles in the Declaration of Helsinki. Ethical approval was provided by the Human Research Ethics Committee at the University of Sydney (2021/122). Total RNA sequencing was performed on RNA isolated from LV tissue with a minimum RIN score of 7. RNA sequencing libraries (Illumina) from PPCM, and normal donor hearts were then profiled using total RNA sequencing (mRNA-Seq) at 30 M raw paired reads/sample on the Illumina NovaSeq 6000. PPCM SHB sequence files were aligned to the GRC human genome build 38 using STAR, and gene count matrices were generated using “Stringtie”, “ballgown”, and the R package “IsoformSwitchAnalyseR” for subsequent analysis in R.

As PPCM is a pregnancy-related condition, data were acquired from female donors and female DCM patients, where possible. Further filtering of GSE141910 was applied to age-match these end-stage PPCM samples, which ranged from 25 to 51 years old, by including donors and DCM patients aged between 20–55 years in our analysis. The SHB dataset contained age-matched donors of both genders.

### 2.2. Data Processing and Identifying Differentially Expressed Genes

The R package edgeR was used for quality control, filtering, and statistical analysis to identify differentially expressed genes (DEGs) between groups [19]. Using edgeR and lmFit (limma), differential log2 transformation and data normalization were applied to the dataset to identify differentially expressed genes (DEGs) between groups. DEGs were identified by comparing DCM to non-failing donors, PPCM (meta) to non-failing donors, and PPCM to DCM. A *p*-value of <0.05 was considered significant and used for subsequent enrichment analysis to ensure that low-level expressed genes that may be biologically relevant were retained. For meta-analysis, data frames generated using the fit2 function were provided to MetaVolcanoR.

### 2.3. DEG Meta-Analysis of PPCM and DCM Patient LVs

To assess the conserved gene expression profiles of PPCM and DCM across independent datasets, random-effects meta-analysis was performed using the R package MetaVolcanoR. Based on the random-effects model, a calculated average log2 expression (effect size), *p*-value and consistency score (signcon) of each gene was obtained. Genes with a *p* < 0.05 and a |signcon| > 2 were considered significant and consistently expressed. The signcon value of 2 indicates the conservation of the expressed gene in two datasets. For meta-PPCM, datasets include all PPCM from GSE141910 and SHB containing 12 PPCM and 35 donors. For meta-DCM, the selected datasets included GSE55296, GSE71613, and GSE141910 from GEO that, respectively, contained RNAseq expression profiles of LV myocardial tissue. GSE55296 contained 13 DCM and 10 non-failing donors. GSE71613 contained 2 DCM and 4 non-failing donors. GSE141910 contained 133 DCM and 136 non-failing donors, which included all males and the remaining females that were excluded from the original analysis. The sample population used for meta-DCM DEG analysis is summarized in Table S1. The meta-DCM with a signcon of 3 genes was applied to verify changes in immune cell population and crucial gene pathways identified in the female DCM cohort. 

### 2.4. Gene Set Enrichment Analysis

We carried out a functional annotation analysis on DEGs using the web-based analytical platform, Database for Annotation, Visualization, and Integrated Discovery (DAVID, v2021, https://david.ncifcrf.gov/; accessed on 18 August 2022). Enrichment in genetic association with annotated gene sets was identified. Annotated gene sets of interest include the genetic association database (GAD) disease class, biological processes (BP) in gene ontology (GO), and Reactome and Kyoto Encyclopedia of Genes and Genomes (KEGG) pathways. 

Additional curated functional analyses using the same DEG list were performed using web-based enrichment analysis tools, Enrichr (https://maayanlab.cloud/Enrichr/ accessed on 18 August 2022) and ToppGene v2021 (https://toppgene.cchmc.org/ accessed on 18 August 2022). Enrichr was used to provide MSigDB hallmark 2020, ChEA 2016, Descartes Cell Types and Tissue 2021, and Azimuth Cell Types 2021. The ToppFun module in the ToppGene Suite was used to detect functional enrichment in the ToppCell Atlas database using annotations derived from the *Transcriptional and Cellular Diversity of the Human Heart* study. Enrichment was confirmed if the Benjamini adjusted *p*-value (or FDR) was <0.05. 

To visualize GSEA findings from the meta-analysis, datasets were combined and normalized using “COMBAT”. Volcano plots and heat maps were plotted using R packages “enhanced volcano”, “gplots”, “RColorBrewer”, and “ggplot2”. Visualizations of systematic gene set enrichment analysis were presented as stem plots generated by inputting DEGs into ShinyGo (Ver 0.77, http://bioinformatics.sdstate.edu/go/; accessed on 16 March 2023).

### 2.5. Cellular Deconvolution

Cibersort was applied to the RNAseq datasets to deconvolute infiltrative inflammatory cells in cardiac tissue by following protocols outlined in [20]. Cibersort is an analytical algorithm used to deconvolute the abundance of 22 immune cell types from a mixed cell population using the relative expression of RNA transcripts measured by RNA sequencing. This algorithm utilizes a core matrix of gene expression data comprising 547 genes and machine learning to robustly deconvolute 22 leukocytes based on the relative gene expression profiles of each cell type. The following immune cell types were identified: naïve B cells, memory B cells, plasma cells (or plasmablasts), cytotoxic T cells (CD8^+^), helper T cells (CD4^+^ naïve, memory, and follicular), regulatory CD4^+^ T cells, gamma-delta T cells, natural killer cells (NK cells; resting and activated), dendritic cells (resting and activated), monocytes, macrophages (M0, M1, and M2), mast cells (resting and activated), eosinophils, and neutrophils. 

GSE183852 was used to create a gene signature matrix derived from the pseudo-bulk gene expression profiles of myocardial cell types, namely cardiomyocytes, epicardium, smooth muscle cells, fibroblasts, endothelium, lymphatic’s, neurons, endocardium, and adipocytes generated in the Seurat R package. This gene signature matrix was then applied using CibersortX to deconvolute cardiac cell-type proportions based on gene expression [21].

### 2.6. Statistical Analysis

Data are expressed as means and standard error measurements, unless otherwise indicated. Comparisons of immune cell abundance between groups were performed using one-way ANOVA with a Tukey post hoc test. A *p*-value of <0.05 was considered statistically significant. Statistical and graphed analyses comparing Cibersort and CibersortX data between groups were generated using GraphPad Prism v9.3.1. All other graphical visuals were generated using R, as described in detail above. 

## 3. Results

### 3.1. Population Information

Samples and datasets included in this study are listed in Table 1. The average age of the two PPCM cohorts was 35 years. The dataset was screened to ensure predominately female donors (from non-cardiac-related deaths), and female DCM patients within a comparable age range were included in our analysis. This resulted in 35 donors with an average age of 41 years, and 33 DCM with an average age of 43 years. The average age of PPCM patients requiring transplant is 10 years younger than DCM patients.

### 3.2. Differential Gene Expression Analysis in Heart Failure

**Figure 1:**
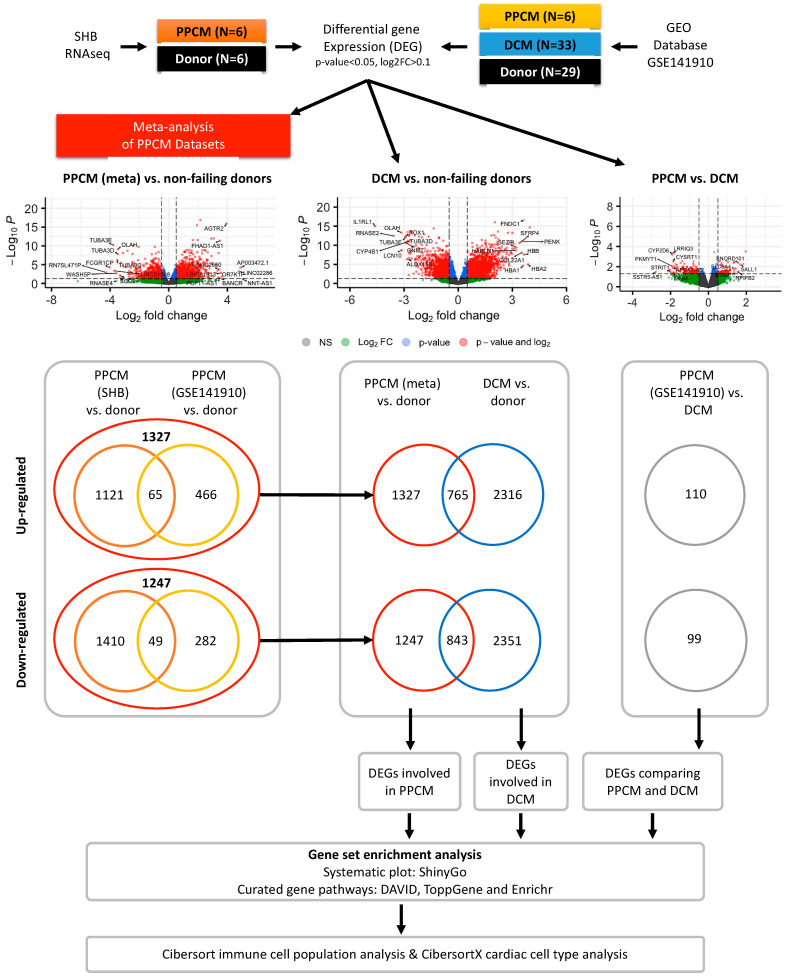
Flow-diagram of differential gene expression profiles in PPCM and DCM heart failure.

To identify differentially regulated genes in disease states, we compared PPCM to donors, DCM to donors, and the overlap between PPCM and DCM (Figure 1). An initial comparison of PPCM from GSE141910 and SHB datasets identified 769 and 2531 genes that were differentially expressed, respectively. Meta-analysis was implemented to identify common DEGs across the two independent PPCM cohorts. Using the meta-DEGs, we identified a total of 1201 conserved genes that were differentially expressed, of which 672 (56%) were upregulated and 529 (44%) were downregulated in both PPCM datasets (Table S2). 

Next, comparison of the gene expression of DCM and donor samples identified 4667 genes that were differentially expressed. Amongst these genes, 2316 (49%) were upregulated and 2351 (51%) were downregulated in DCM (Table S3). The DCM-conserved DEGs were verified using a meta-analysis of three separate datasets (Table S1), which consisted of 4674 upregulated genes and 4947 downregulated genes (Table S4). We also identified the DEGs that overlap between PPCM and female DCM resulting in 1608 genes (765 upregulated and 843 downregulated). 

Finally, a comparison of PPCM and DCM using dataset GSE141910 identified a total of 209 differentially regulated genes, with 110 being upregulated and 99 being downregulated. Of note is the fact that the SHB dataset did not comprise DCM samples, thereby limiting comparison. No significant enrichment in gene set pathways and processes were identified in DAVID, ToppCell, and Enrichr for this comparison. The DEGs described above are summarized in Figure 1.

### 3.3. Enrichment Analysis of Heart Failure DEGs

To investigate the processes and pathways associated with genes that were differentially expressed in each disease state, systematic functional and gene set enrichment analysis using DAVID, ToppGene, and Enrichr was performed, and notable terms were curated in Tables S5–S8. For each comparison, upregulated and downregulated gene sets were analyzed independently. 

**Figure 2:**
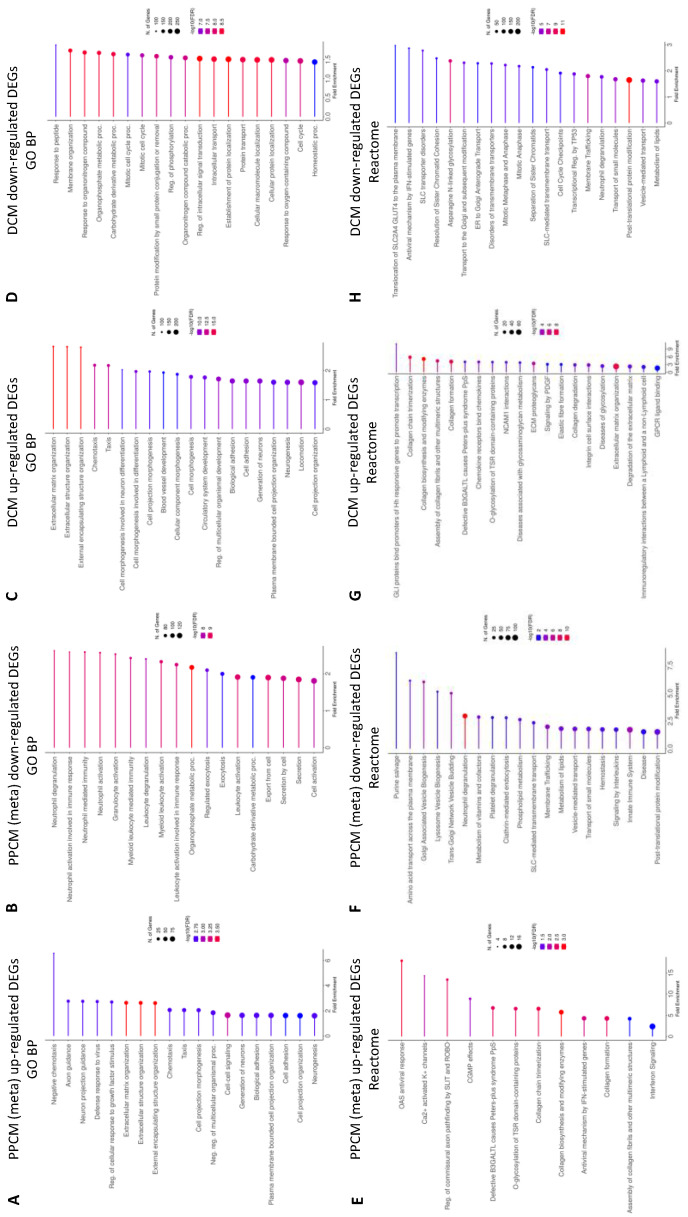
Enriched pathways in PPCM (meta) and DCM. The top 20 pathways with FDR < 0.05 are sorted by fold enrichment. (**A***–***D**) shows enriched gene ontology biological processes (GO BP), and (**E***–***H**) shows enriched Reactome pathways.

A summary of the top 20 significantly (where available) up- or downregulated systematic GO BP and Reactome pathways in PPCM and DCM is displayed in Figure 2. Significantly enriched pathways and processes will be described in detail in the proceeding sections.

### 3.4. Enrichment Analysis Comparing PPCMs and NF Donors

A systematic analysis of genes with higher expression levels in PPCM showed metabolic (*p*-value = 3.02 × 10^4^), cardiovascular (*p*-value = 2.12 × 10^7^), and immune (*p*-value = 1.38 × 10^2^) enrichment in the disease class. Pathway analysis showed Reactome enrichment associated with matrix remodeling and viral responses, but no significant enrichment was found in KEGG. GO analysis identified terms related to the extracellular matrix organization, viral response, calcium, and heparin binding were significantly enriched. Hallmark terms identified enrichment in interferon alpha (*p*-value = 1.08 × 10^4^) and interferon gamma (*p*-value = 5.01 × 10^5^) responses. Among the curated cell lineage enrichment pathways, ToppCell cell atlas analysis rendered significant enrichment in vascular smooth muscle cells (*p*-value = 1.96 × 10^16^), fibroblasts (*p*-value = 9.13 × 10^10^), and lymphocytes (*p*-value = 1.07 × 109) from the LV. Descartes analysis of tissue-specific cell types produced the following enriched *p*-values: lymphoid (*p*-value = 5.38 × 10^7^), stromal (*p*-value = 4.11 × 10^2^), and Schwann (*p*-value = 1.12 × 10^2^) cells in the heart, and lymphoid cells in the placenta (*p*-value = 2.99 × 10^6^). Azimuth cell-type analysis shows terms that are strongly related to NK and CD4^+^/8^+^ T cells. For summary, see Table S5 and Figures S1–S3.

Systematic analysis of genes with reduced expression in PPCM revealed significant enrichment in pathway analysis but no associations with disease classes or GO BP were identified. For Reactome, these genes were significantly enriched for terms related to metabolism (*p*-value = 1.03 × 10^4^), immune system (*p*-value = 2.50 × 10^5^), and the Golgi complex (*p*-value = 2.38 × 10^5^). A similar enrichment of metabolic terms (*p*-value = 1.74 × 10^4^) was identified in KEGG. Curated cell lineage analysis using the ToppGene cell atlas analysis found enriched populations of macrophage (*p*-value = 6.78 × 10^29^), and endothelial cells (*p*-value = 3.71 × 10^12^) derived from the LV chamber were associated with these genes. For Descartes cell-type analysis, we found enriched myeloid cells in the heart (*p*-value = 3.06 × 10^3^), but no significant enrichment in Azimuth cell types were detected. These genes were significantly enriched for protein transport, fatty acid metabolism, adipogenesis, and immune signaling in Hallmark gene sets. For summary, see Table S6 and Figures S4–S8.

Further analysis of downregulated Reactome ‘metabolism’ and ‘immune system’ gene sets were assessed by inputting the resulting significant gene list into DAVID. The ‘metabolism’ gene set saw the enrichment of the metabolism of lipids (R-HSA-556833, *p*-value = 1.88 × 10^30^), phospholipid (R-HSA-1483257, *p*-value = 8.42 × 10^15^), and amino acids (R-HSA-71291, *p*-value = 2.23 × 10^10^). The ‘immune system’ gene set resulted in the enrichment of the innate immune system (R-HSA-168249, *p*-value = 1.80 × 10^63^), adaptive immune system (R-HSA-1280218, *p*-value = 1.48 × 10^29^), cytokine signaling in the immune system (R-HSA-1280215, *p*-value = 1.48 × 10^29^), and signaling by interleukins (R-HSA-449147, *p*-value = 9.06 × 10^24^) as top significant terms. 

### 3.5. Enrichment Analysis Comparing DCMs and NF Donor Controls

Systematic analysis of the genes with higher expression in DCM established that the majority were associated with cardiovascular (*p*-value = 1.07 × 10^4^) and immune (*p*-value = 2.69 × 10^4^) disease classes. Both KEGG and Reactome pathways revealed significantly enriched extracellular matrix remodeling and immune signaling terms. The latter is supported by the Hallmark gene set enrichment for interferon alpha (*p*-value = 1.37 × 10^8^), interferon gamma (*p*-value = 3.80 × 10^8^), and IL2/STAT5 signaling (*p*-value = 2.41 × 10^2^). Curated cell lineage analysis using ToppGene revealed genes for lymphocytes, vascular smooth muscle cells, neuronal, and fibroblasts in the LV chamber were significantly enriched in DCM. A similar enrichment of lymphoid (*p*-value = 4.32 x 10^20^), stromal (*p*-value = 1.84 × 10^5^), Schwann (*p*-value = 5.43 × 10^4^), and vascular endothelial (*p*-value = 9.74 × 10^3^) cells was detected in the Descartes cell-type analysis. The Azimuth analysis shows that lymphoid cells may be associated with natural killer cells, dendritic cells, CD8^+^/CD4^+^ T cells and naïve B cells. For summary, see Table S7 and Figures S9–S13.

Genes with reduced expression in DCM overlapped with metabolic (*p*-value = 8.39 × 10^3^) and cardiovascular (*p*-value = 5.34 × 10^3^) disease classes. These genes were significantly enriched for protein modification in Reactome and GO BP, and cellular development in KEGG. Cell lineage analysis using ToppGene revealed the enrichment of gene sets associated with endothelial, macrophage, and fibroblasts, but no significant Descartes and Azimuth cell types were identified. For Hallmark, multiple terms relating to oxidative stress, metabolism, inflammatory response gene sets were enriched. See Table S8 for summary. Enrichment in similar processes and pathways was found in DCM meta-analysis comparing all remaining DCM and donors from GSE141910, GSE55296, and GSE71613 (Tables S9 and S10). 

### 3.6. Infiltration of Immune Cell Populations in the Failing Heart

The term ‘immune’ was significantly enriched in the gene-associated disease (GAD) in both PPCM and DCM. To determine the potential contribution of infiltrative immune cells in the heart, Cibersort was used to estimate the abundance of immune cells based on the deconvoluted immune signatures of PPCM, DCM, and non-failing donors. 

**Figure 3:**
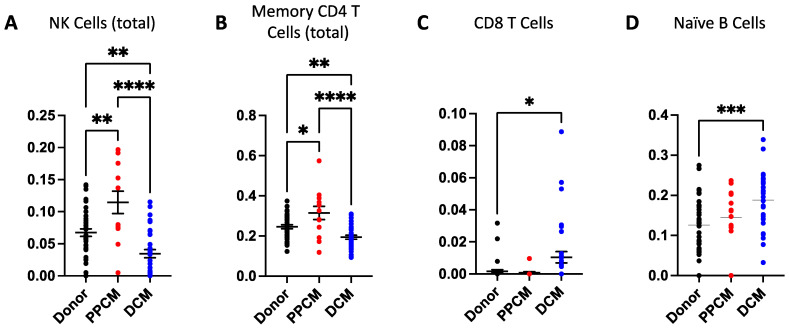
Infiltrative immune cell populations in diseased and donor hearts derived from lymphoid lineages. Elevated natural killer (**A**) and memory CD4^+^ T cell (**B**) populations were identified in PPCM. In contrast, DCM had reduced levels of natural killer (**A**) and memory CD4^+^ T cell (**B**) populations. DCM patients also had elevated levels of CD8^+^ T Cells (**C**) and naïve b cells (**D**). Significance is denoted as * *p* < 0.05, ** *p* < 0.01, *** *p* < 0.001, **** *p* < 0.0001. Data are presented as mean and standard error measurements.

**Figure 4:**
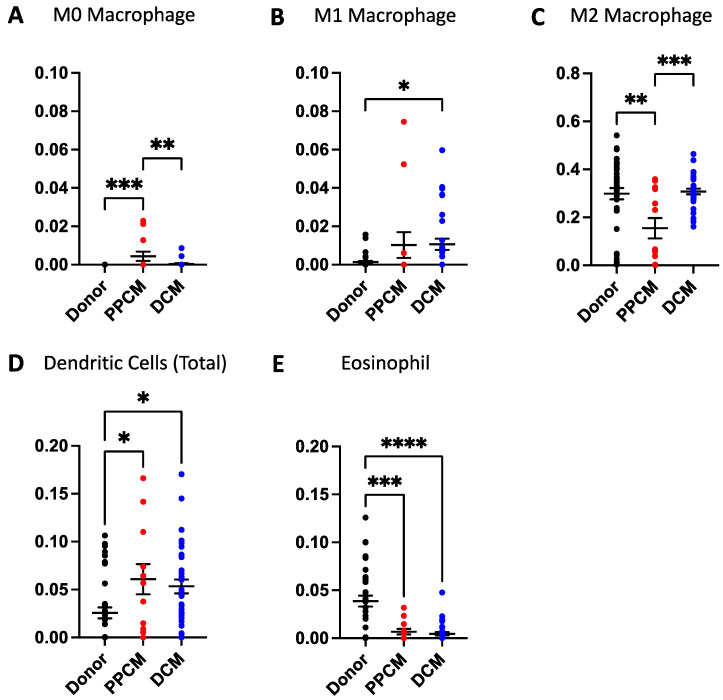
Infiltrative immune cell populations in diseased and donor hearts derived from myeloid lineages. (**A**) M0 macrophage, (**B**) M1 macrophage, and (**C**) M2 macrophage populations differed in select disease states. (**D**) Both dendritic cells and (**E**) eosinophils both significantly differed in PPCM and DCM compared to donors. Significance is denoted as * *p* < 0.05, ** *p* < 0.01, *** *p* < 0.001, **** *p* < 0.0001. Data are presented as mean and standard error measurements.

Immune cell fractions from both lymphoid (Figure 3) and myeloid (Figure 4) lineages were compared across PPCM, DCM, and non-failing donor LV myocardial tissue. Among the 22 immune cell types, 21 cell types were detected in one or more samples with the exception of memory B cells. Of the 21 detected cell types, PPCM and DCM significantly differed to donor heart samples in six and seven immune cell types, respectively. These include myeloid-derived cell types (M0, M1, and M2 macrophages and eosinophil and dendritic cells) and lymphoid-derived cell types (naïve B cell, CD8^+^ T cell, memory CD4^+^ T cell, and NK cells). 

In PPCM, six immune cell types were significantly expressed relative to donor levels. NK cell (Figure 3A) and Memory CD4^+^ T cell (Figure 3B) populations were higher in the PPCM relative to donors. Similar increases were identified for M0 macrophages (Figure 4A) and dendritic cell populations (Figure 4D). In comparison, M2 macrophages were effectively halved, and eosinophils were reduced by approximately five-fold in PPCM.

In DCM, seven immune cell types significantly differed from donor levels. M1 macrophages (Figure 4B) and total dendritic cells (Figure 4D) were significantly enriched in DCM with significant contributions from the resting dendritic cell population. In contrast, eosinophils (Figure 4E) were found to be present at lower levels. Amongst the immune cells derived from lymphoid origins, naïve B cells and cytotoxic CD8^+^ T cells were also enriched (Figure 4C,D), while the memory CD4^+^ T and NK cell populations (Figure 4A,B) were reduced in DCM compared to donor controls. The changes in immune cell populations found in the female DCM population were verified in the meta-DCM population, suggestive of a common disease pathway irrespective of age and sex (Table S11).

**Figure 5:**
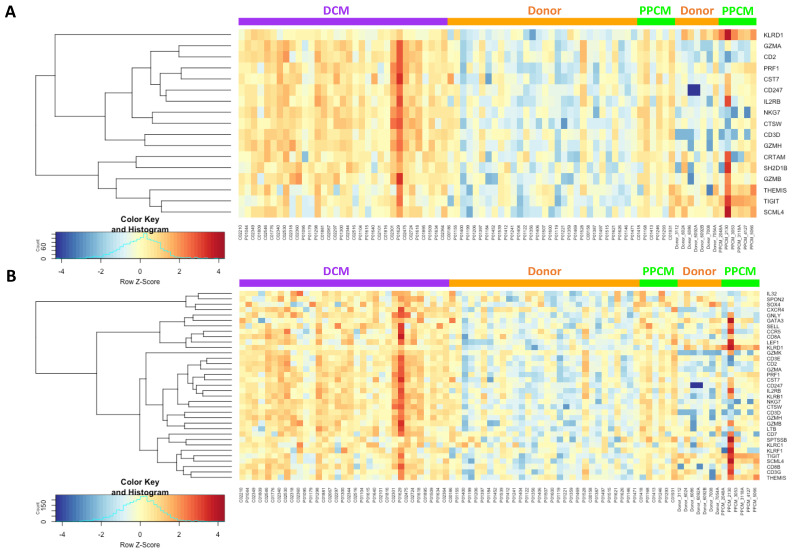
Heatmaps showing upregulated cytotoxic T cell genes in (**A**) PPCM and (**B**) DCM. DCM (purple), PPCM (green), and donor controls (orange).

Next, we evaluated the number of cytotoxic T cell genes upregulated in PPCM and DCM. We identified 17 genes in PPCM (Figure 5A) and 35 genes in DCM (Figure 5B), with an overlap in 15 of these cytotoxic T cell genes. In PPCM, class-I MHC-restricted T-cell-associated molecule (*CRTAM*) and SH2 domain containing 1B (*SH2D1B*) genes were uniquely expressed. In DCM, groupings of killer lectin receptor (*KLR*; *KLRB1, KLRC1*, and *KLRF1*) and granzyme (*GZM*; *GZMA, GZMB*, and *GZMK*) genes are upregulated to a greater extent compared to PPCM and donors. 

### 3.7. Cell Types Annotated by DEG Signatures

**Figure 6:**
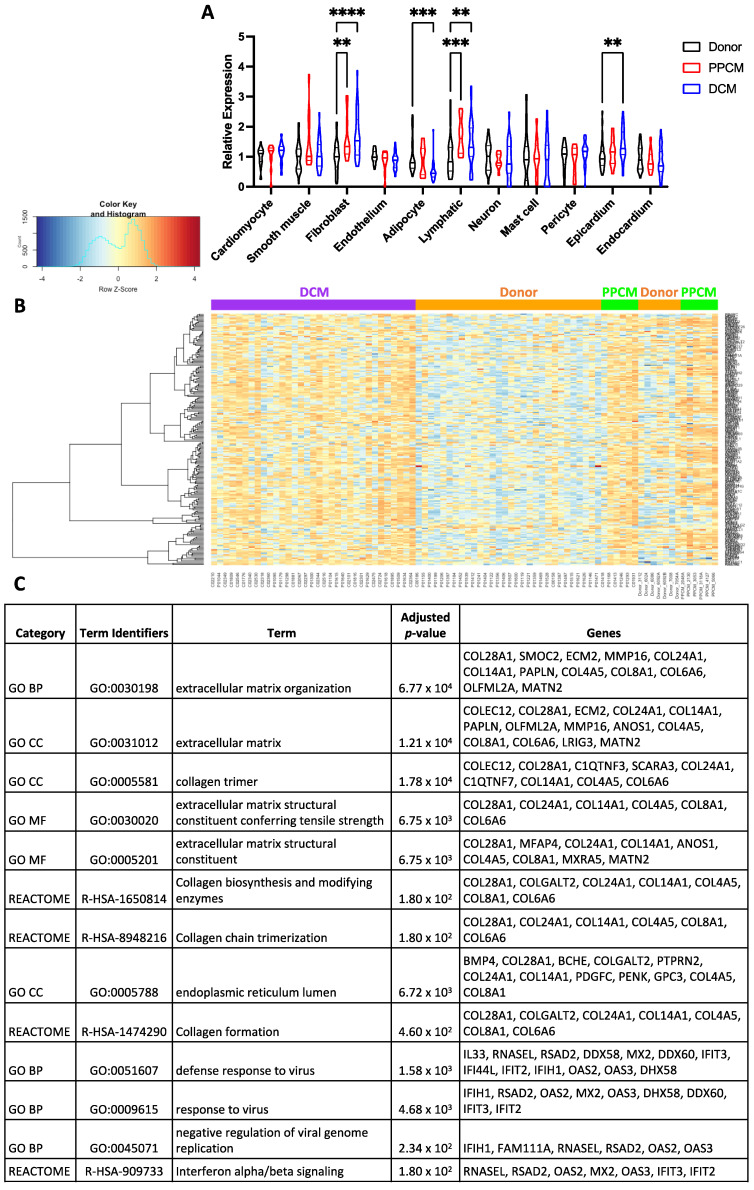
Cell types in the LV myocardium of PPCM and DCM. (**A**) Cell-type composition deconvoluted from PPCM and DCM gene expression profiles using CibersortX. PPCM and DCM data are normalized to donor values. (**B**) Heatmap summarizing DEGs expressed in fibroblasts in PPCM (green), DCM (purple), and donors (orange). (**C**) Enrichment analysis summarizing the representative upregulated pathways and processes from the genes in (**B**). Significance is denoted as ** *p* < 0.01, *** *p* < 0.001, and **** *p* < 0.0001. Data presented as mean and standard error measurements.

To assess non-immune cell types, the CibersortX algorithm was applied to infer the relative cell-type gene expression profiles from bulk tissue transcriptome. Reference cell-specific gene signatures were categorized and annotated in the single-cell RNAseq dataset GSE183852 [22]. The abundance of 11 cell types were inferred from donor, PPCM and DCM samples (Figure 6A). Overall, four cell-type populations significantly differed from donors, namely fibroblasts, adipocytes, lymphatic, and epicardial cells. In DCM, increased expression was observed in three of the four cell types, apart from adipocytes. In PPCM, fibroblasts and lymphatic cell populations were significantly larger than donors. 

A high-resolution expression of the significant PPCM meta-analytic genes imputed by CibersortX revealed that amongst the cell types, fibroblasts showed the most consistent gene expression across disease and donor groups (Figure 6B). The fibroblast-specific imputed genes were then examined using DAVID, which demonstrated strong associations with processes involved with the extracellular matrix, endoplasmic reticulum, and immune response. 

## 4. Discussion

In this study, we compared the myocardial gene expression in PPCM, DCM, and non-failing donors to reveal several important findings. First, a subset of immune cells detected in the PPCM myocardium are distinct from DCM. Second, the downregulated PPCM DEGs overlap with unique gene sets involved in the immune system and the Golgi vesicles. These pathways appeared distinct from the transcriptome found in DCM and donor. Finally, DCM gene sets were correlated with a wider array of pathways, including metabolism, extracellular matrix organization, cell signaling, protein modification, and ubiquitination pathways, which are represented to a lesser extent in PPCM. Overall, these specific transcriptomic signatures and cellular expression provide insights into the pathophysiology of end-stage cardiomyopathy that has not been previously reported. Importantly, the information gained from this study may reveal key physiological changes that differentiate PPCM from the more common form of systolic heart failure, DCM.

**Immune cell signatures of PPCM and DCM**

PPCM is diagnosed around the time of delivery when maternal cellular immunity is returning to baseline from a physiologically downregulated state that safeguards fetal tolerance. A recent study on PPCM peripheral blood shows higher levels of regulatory T cells but reduced NK cells within the 6-week post-partum period, with levels normalizing within six months when compared to blood samples from non-diseased pregnant women [11]. The same study showed that a similarly reduced NK cell profile was present in women with recent onset (<6 months) non-ischemic cardiomyopathy compared to women who were not pregnant. Our findings in DCM—a prolonged form of recent onset cardiomyopathy—similarly identified reduced NK cell levels in tissue, and also compared to non-pregnant healthy women. These immune changes may serve as a useful biomarker for non-ischemic heart disease. 

Our study also observed significant changes in memory CD4^+^ T cells, CD8^+^ T cells, and naïve B cell populations in DCM, which were independently verified by Fang et al. (2022) [23]; however, these populations were not present in the blood of recent-onset cardiomyopathy or PPCM [11]. This suggests that the B and T cell responses identified in this study may not be systemic (i.e., specific to the myocardium), and the result of prolonged cardiac inflammation may be contributing to the end-stage DCM pathogenesis. Previously, murine models of severe DCM were generated by PD1 (programmed cell death protein 1) knockout, normally expressed by activated T cells and promotes B cell differentiation, resulting in high levels of circulating cardiomyocyte-specific IgG autoantibodies [24]. Our gene set analysis found upregulated ‘PD-1 signaling’ overlapping with ‘co-stimulation by the CD28 family’ in DCM but not in PPCM patients. Productive CD8^+^ T cell co-stimulation is seemingly crucial for restoring the CD8^+^ T cell adaptive response that becomes exhausted during chronic infections in the heart [25] and in cancers [26]. DCM is further complicated by the presence of naïve and memory B cell levels, which were both strongly correlated with increased circulating Troponin I, which is indicative of myocardial injury [27]. Injury signals the recruitment of pro-inflammatory infiltrates, such as CD8^+^ T cells and M1 macrophages, and mediates T cell activation [16], which are consistent with the estimated DCM immune cell scores and cell-lineage gene expression changes in this study. Together, end-stage DCM is complicated by a multitude of pro-inflammatory changes, with the most evident being T-cell-mediated cytotoxicity, which clearly distinguishes it from early stage disease.

The present study also finds evidence of enriched cytotoxic T cell genes in PPCM and DCM. For example, the unique PPCM genes, *CRTAM* and *SH2D1B*, are, respectively expressed on CD4^+^/CD8^+^ T cells and the effector molecule of NK cells, and may play a role in the local immune response. *CRTAM* expressing T cells secrete interferon gamma and activate granzyme B (*GZMB*), which induces cardiac apoptosis [28]. Concurrently, we also identified an upregulated interferon gamma response, which is a cytokine associated with the pro-inflammatory Th1 cell subtype. This pro-inflammatory subtype was previously detected at slightly higher levels in the serum of newly diagnosed PPCM patients [29] and in DCM [14]. These pathways and cytokines serve as indicators of immune regulation that was not otherwise identified in end-stage PPCM. As such, the pro-inflammatory responses may be more evident in DCM with its increased cytotoxic CD8^+^ T Cell and M1 macrophage populations, but it is not totally absent from PPCM.

In PPCM, a downregulation of myeloid cell genes appears to correlate with a reduction in the M2 macrophage population. DCM also shows a similar but not significant change in the M2 macrophage population. The alternatively activated M2 macrophage population carries anti-inflammatory properties and promotes adaptive remodeling in response to mechanical stress [3]. Macrophages are classically described in the context of the M1/M2 polarization axis that compares the ratio between the two macrophage phenotypes, and possibly contributes to the observed differences between PPCM and DCM. Irrespective, the M2 phenotype is not well understood in cardiac immunity, although one study had associated M2 with collagen formation and ventricular remodeling in DCM [15]. 

Taken together, PPCM does not seem to display an overtly inflammatory phenotype, and suggests that another process may be involved in disease manifestation and progression. Unique enriched terms that clearly distinguish PPCM from DCM were identified when comparing downregulated DEGs-associated pathways. Of interest are the terms associated with metabolism and Golgi biogenesis.

**Metabolic pathways in PPCM and DCM**

In PPCM, several metabolic terms were identified to be enriched in the significant and conserved meta-analytic genes, including ‘metabolic pathways’, ‘metabolism’, ‘fatty acid metabolism’, ‘adipogenesis’, ‘heme metabolism’, ‘xenobiotic metabolism’, ‘cholesterol homeostasis’, and ‘insulin signaling pathway’. While DCM also displayed a similar enrichment of metabolic pathways, the regulation of the cellular metabolism via MTOR, MAPK, and FOXO signaling, as well as associated ‘insulin resistance’ and ‘glycolysis’ suggests differences in underlying pathogenesis. The dysregulation of FOXO, MAPK, and MTOR signaling pathways was reported to play important roles in glucose metabolism leading to insulin-resistance-related metabolic disorders found in cancer [30,31,32], but their roles in PPCM and DCM are not well defined.

The heart is a metabolically demanding organ that can utilize various forms of energy substrates depending on the availability and activity undertaken. Under basal physiological conditions, 70% of the energy substrate is supplied by fatty acids and the remaining is supplemented by glucose [33]. During stress and disease, the heart can quickly switch sources of energy [34]. This was previously shown in PET imaging of DCM patients with impaired fatty acid uptake and increased rates of glucose uptake [35]. In PPCM, aberrant regulation of lipid metabolism was also identified, and this was found in induced pluripotent stem-cell-derived cardiomyocytes from two PPCM patients diagnosed within a week after delivery [36]. The PPCM study concluded that lipid metabolism was inhibited, but more surprisingly, the subsequent switch to anerobic glycolysis was blunted. This was not the case in healthy stem cells, which demonstrated metabolic plasticity using different energy sources. Furthermore, unlike other metabolically demanding organs, such as the liver, the heart does not synthesize fatty acids, so it acquires them in the form of cholesterol from the circulation under otherwise normal conditions [37]. Enrichment in ‘cholesterol homeostasis’ downregulated in PPCM suggests that altered lipid homeostasis may contribute to the reduced uptake. It was hypothesized that the reduced utilization of fatty acids and lipids not only impedes energy reserves resulting in a mismatch in demand and supply, but also the accumulation of fatty acid derivatives may lead to the lipotoxicity that was demonstrated in various forms of heart disease, including DCM [34]. 

Notably, our study also identified enrichment in ‘trans-Golgi network vesicle budding’ and ‘Golgi associated vesicle biogenesis’. The formation of golgi vesicles is critical in the processing and transport of proteins and lipids to various parts of the cell, and these dynamic functions are intricately interlinked to lipid metabolism [38]. Prior studies of Golgi vesicles isolated from hearts were strongly associated with cardiac disorders, including pulmonary hypertension, arrhythmias, and DCM [39]. In DCM, Golgi vesicles were smaller but present in greater numbers, which correlated with the LV ejection fraction. While we are the first to describe Golgi and metabolic enrichment terms in PPCM, the mechanistic underpinnings of lipid metabolism from the Golgi complex to its utilization as an energy substrate remains to be elucidated.

Together, blunting the heart’s ability to switch between metabolic sources was considered a potential key factor for PPCM susceptibility [36]. This limitation may be underpinned by aberrant Golgi dynamics contributing to ongoing pathogenesis. Further characterization of metabolic function and the transportation of newly synthesized proteins and lipids would provide significant insights into DCM and PPCM metabolism, and remains a limitation of this study that requires further investigations.

**Myocardial fibrosis is common to PPCM and DCM**

In the context of upregulated DEGs in PPCM, we identified enrichment in extracellular matrix organization, viral response, calcium binding, heparin binding, and inflammatory immune response associated with the adaptive immune system. These enriched gene sets appear to be common physiologic processes in systolic heart failure because the same terms were identified in DCM. Unsurprisingly, changes to extracellular matrix organization, collagen biosynthesis and matrix constituents impart functional consequences to the heart by modifying myocardial stiffness. This was previously characterized using picrosirius red staining of end-stage PPCM and DCM myocardial samples derived from the Sydney Heart Bank [40]. In this study, the authors showed a similar 5-fold increase in total fibrosis in PPCM and DCM with a secondary finding of increased calcium sensitivity due to impaired PKA-mediated phosphorylation, possibly to compensate for the myocardial stiffening. Intriguingly, gene set enrichment revealed downregulated ‘post-translational protein modification’ concurring with PKA hypo-phosphorylation in DCM but not in PPCM [40]. Our pseudo-single cell analysis showed increased fibroblast cell numbers in both heart failure groups coinciding with increased fibrosis that was confirmed in DCM patients using late gadolinium imaging and histopathology staining [41]. Furthermore, immune cells, which respond to mechanical stress, elicited by changes in myocardial stiffness and extracellular matrix remodeling, can stimulate pro-inflammatory fibrogenic signaling [42]. 

It remains unknown whether the inflammatory response and the associated myocardial remodeling in human hearts is due to an undiagnosed viral infection. Evidence presented from mice models, such as the myosin binding protein C mutant inducing DCM [43] and desmocollin-2 overexpression that induced arrhythmogenic cardiomyopathy (ACM) [44], reveals direct links between genetic mutations and pro-inflammatory responses causing cardiac fibrosis independent of acquired immune activators. It is possible that the enhanced inflammatory response can be activated by myocyte damage, which is where these mutations are localized, that then initiates a fibrotic cascade and fibrofatty deposits [3,43,44]. Furthermore, the inflammatory and fibrotic responses common to systolic heart failure may be derived from diverse genotype-specific transcriptomic profiles [45]. Sielemann et al. examined four different gene mutations that cause DCM or ACM, and showed that distinct gene sets and pathways can produce similar end-stage phenotypes [45]. These findings suggest that there are multiple routes to disease pathogenesis, and understanding the distinct cellular mechanism, such as that presented in our paper, will be useful in understanding underlying disease processes.

The present findings should be interpreted accordingly. Firstly, myocardial gene expression in healthy postpartum women was not available to identify pregnancy-associated physiology. Phasic adaptations in innate and adaptive immunity during pregnancy have previously been reported from peripheral blood but are not available from heart tissue [11]. Secondly, age- and sex-matched donor and heart failure samples are restricted by the rarity of PPCM. The limited availability of tissue sample directly influences the effect size, overall number of statistically significant differentially regulated genes, and secondary validation of key genes by alternative methods. However, immune and gene set analysis of expanded DCM and donor cohort to include males revealed no further differences.

## 5. Conclusions

In conclusion, RNAseq analysis revealed the unique gene expression profiles of PPCM and DCM from the human LV samples. Our study identifies several differences between PPCM and DCM that contribute to end-stage disease pathology. Both categories of heart failure display similar enrichment in metabolic pathways and extracellular matrix remodeling associated with late-stage systolic heart failure. The enrichment of Golgi-associated vesicle terms in PPCM hearts suggests a unique protein/lipid-mediated pathophysiology. Furthermore, immune changes in PPCM are not present to the same extent as DCM, which was associated with pronounced pro-inflammatory and cytotoxic T cell activity. Despite the limited number of patients involved in this study, it discloses novel pathways that are features of end-stage heart failure and potential mechanisms of disease between PPCM and DCM.

## Figures and Tables

**Table 1:** Demographics of human donor, DCM, and PPCM samples.

		Donor	PPCM	DCM
SHB	N	6	6	-
	Age (years)	34.8 ± 8.0	34.2 ± 6.9	-
	Female (%)	67	100	-
GSE141910	N	29	6	33
	Age (years)	47.4 ± 9.8	34.7 ± 10.3 *	42.5 ± 10.9
	Female (%)	100	100	100
Total N		35	12	33

* Average age for PPCM was significantly lower than donors (*p*-value = 2.38 × 10^2^). No other differences in age were detected in each cohort.

## Data Availability

Datasets used in this study were acquired from the gene expression omnibus; GSE141910, GSE71613 GSE55296, and GSE183852. Sydney Heart Bank dataset can be made available up reasonable request.

## Supplementary Materials

The following supporting information can be downloaded at: https://www.mdpi.com/article/10.3390/jcdd10050188/s1. Figure S1: heatmap of interferon alpha response in PPCM. Figure S2: heatmap of interferon gamma response in PPCM. Figure S3: heatmap of lymphoid cell in heart in PPCM. Figure S4: heatmap of metabolic pathways in PPCM. Figure S5: heatmap of fatty acid metabolism in PPCM. Figure S6: heatmap of adipogenesis in PPCM. Figure S7: heatmap of phospholipid metabolism in PPCM. Figure S8: heatmap of immune system reactome pathway in PPCM. Figure S9: heatmap of costimulation by CD28 family in DCM. Figure S10: heatmap of PD1 signaling in DCM. Figure S11: heatmap of immune response in DCM. Figure S12: heatmap of Th1 and Th2 cell differentiation upregulated in DCM. Figure S13: heatmap of Th17 cell differentiation upregulated in DCM. Table S1: summary of GEO datasets for DCM meta-analysis. Table S2: meta-analysis of PPCM DEGs from GSE141910 and SHB. Table S3: DCM (females) vs. Donor DEGs from GSE141910. Table S4: meta-analysis of DCM DEGs from GSE141910, GSE55296 and GSE71613. Table S5: enrichment analysis of upregulated DEGs in PPCM (meta). Table S6: enrichment analysis of downregulated DEGs in PPCM (meta). Table S7: enrichment analysis of upregulated DEGs in DCM. Table S8: enrichment analysis of downregulated DEGs in DCM. Table S9: gene set enrichment analysis of upregulated DEGs in meta-DCM using DAVID. Table S10: gene set enrichment analysis of downregulated DEGs in meta-DCM using DAVID. Table S11: immune cell populations in the DCM and donor heart from GSE55296, GSE71613, and GSE141910 (male samples).

## References

[1] Arany Z., Elkayam U. (2016). Peripartum Cardiomyopathy. Circulation.

[2] Li A., Campbell K., Lal S., Ge Y., Keogh A., Macdonald P.S., Lau P., Lai J., Linke W.A., Van der Velden J. (2022). Peripartum cardiomyopathy: A global effort to find the cause and cure for the rare and little understood disease. Biophys. Rev..

[3] Frantz S., Falcao-Pires I., Balligand J.L., Bauersachs J., Brutsaert D., Ciccarelli M., Dawson D., de Windt L.J., Giacca M., Hamdani N. (2018). The innate immune system in chronic cardiomyopathy: A European Society of Cardiology (ESC) scientific statement from the Working Group on Myocardial Function of the ESC. Eur. J. Heart Fail..

[4] Goli R., Li J., Brandimarto J., Levine L.D., Riis V., McAfee Q., DePalma S., Haghighi A., Seidman J.G., Seidman C.E. (2021). Genetic and Phenotypic Landscape of Peripartum Cardiomyopathy. Circulation.

[5] Ware J.S., Li J., Mazaika E., Yasso C.M., DeSouza T., Cappola T.P., Tsai E.J., Hilfiker-Kleiner D., Kamiya C.A., Mazzarotto F. (2016). Shared Genetic Predisposition in Peripartum and Dilated Cardiomyopathies. N. Engl. J. Med..

[6] Gerull B., Klaassen S., Brodehl A., Erdmann J., Moretti A. (2019). The Genetic Landscape of Cardiomyopathies. Genetic Causes of Cardiac Disease.

[7] Sliwa K., Petrie M.C., van der Meer P., Mebazaa A., Hilfiker-Kleiner D., Jackson A.M., Maggioni A.P., Laroche C., Regitz-Zagrosek V., Schaufelberger M. (2020). Clinical presentation, management, and 6-month outcomes in women with peripartum cardiomyopathy: An ESC EORP registry. Eur. Heart J..

[8] Sliwa K., Mebazaa A., Hilfiker-Kleiner D., Petrie M.C., Maggioni A.P., Laroche C., Regitz-Zagrosek V., Schaufelberger M., Tavazzi L., van der Meer P. (2017). Clinical characteristics of patients from the worldwide registry on peripartum cardiomyopathy (PPCM): EURObservational Research Programme in conjunction with the Heart Failure Association of the European Society of Cardiology Study Group on PPCM. Eur. J. Heart Fail..

[9] Moulig V., Pfeffer T.J., Ricke-Hoch M., Schlothauer S., Koenig T., Schwab J., Berliner D., Pfister R., Michels G., Haghikia A. (2019). Long-term follow-up in peripartum cardiomyopathy patients with contemporary treatment: Low mortality, high cardiac recovery, but significant cardiovascular co-morbidities. Eur. J. Heart Fail..

[10] Halliday B.P., Wassall R., Lota A.S., Khalique Z., Gregson J., Newsome S., Jackson R., Rahneva T., Wage R., Smith G. (2019). Withdrawal of pharmacological treatment for heart failure in patients with recovered dilated cardiomyopathy (TRED-HF): An open-label, pilot, randomised trial. Lancet.

[11] McTiernan C.F., Morel P., Cooper L.T., Rajagopalan N., Thohan V., Zucker M., Boehmer J., Bozkurt B., Mather P., Thornton J. (2018). Circulating T-Cell Subsets, Monocytes, and Natural Killer Cells in Peripartum Cardiomyopathy: Results From the Multicenter IPAC Study. J. Card. Fail..

[12] Koczo A., Marino A., Jeyabalan A., Elkayam U., Cooper L.T., Fett J., Briller J., Hsich E., Blauwet L., McTiernan C. (2019). Breastfeeding, Cellular Immune Activation, and Myocardial Recovery in Peripartum Cardiomyopathy. JACC Basic Transl. Sci..

[13] Zeng Z., Wang K., Li Y., Xia N., Nie S., Lv B., Zhang M., Tu X., Li Q., Tang T. (2017). Down-regulation of microRNA-451a facilitates the activation and proliferation of CD4^+^ T cells by targeting Myc in patients with dilated cardiomyopathy. J. Biol. Chem..

[14] Efthimiadis I., Skendros P., Sarantopoulos A., Boura P. (2011). CD4+/CD25+ T-Lymphocytes and Th1/Th2 regulation in dilated cardiomyopathy. Hippokratia.

[15] Nakayama T., Sugano Y., Yokokawa T., Nagai T., Matsuyama T.A., Ohta-Ogo K., Ikeda Y., Ishibashi-Ueda H., Nakatani T., Ohte N. (2017). Clinical impact of the presence of macrophages in endomyocardial biopsies of patients with dilated cardiomyopathy. Eur. J. Heart Fail..

[16] Rurik J.G., Aghajanian H., Epstein J.A. (2021). Immune Cells and Immunotherapy for Cardiac Injury and Repair. Circ. Res..

[17] Wu J., Sun P., Chen Q., Sun Y., Shi M., Mang G., Yu S., Zheng Y., Li Z., Sun M. (2019). Metabolic reprogramming orchestrates CD4+ T-cell immunological status and restores cardiac dysfunction in autoimmune induced-dilated cardiomyopathy mice. J. Mol. Cell. Cardiol..

[18] Afanasyeva M., Georgakopoulos D., Belardi D.F., Bedja D., Fairweather D., Wang Y., Kaya Z., Gabrielson K.L., Rodriguez E.R., Caturegli P. (2005). Impaired up-regulation of CD25 on CD4+ T cells in IFN- γ knockout mice is associated with progression of myocarditis to heart failure. Proc. Natl. Acad. Sci. USA.

[19] Robinson M.D., McCarthy D.J., Smyth G.K. (2010). edgeR: A Bioconductor package for differential expression analysis of digital gene expression data. Bioinformatics.

[20] Newman A.M., Liu C.L., Green M.R., Gentles A.J., Feng W., Xu Y., Hoang C.D., Diehn M., Alizadeh A.A. (2015). Robust enumeration of cell subsets from tissue expression profiles. Nat. Methods.

[21] Newman A.M., Steen C.B., Liu C.L., Gentles A.J., Chaudhuri A.A., Scherer F., Khodadoust M.S., Esfahani M.S., Luca B.A., Steiner D. (2019). Determining cell type abundance and expression from bulk tissues with digital cytometry. Nat. Biotechnol..

[22] Koenig A.L., Shchukina I., Amrute J., Andhey P.S., Zaitsev K., Lai L., Bajpai G., Bredemeyer A., Smith G., Jones C. (2022). Single-cell transcriptomics reveals cell-type-specific diversification in human heart failure. Nat. Cardiovasc. Res..

[23] Fang C., Lv Z., Yu Z., Wang K., Xu C., Li Y., Wang Y. (2022). Exploration of dilated cardiomyopathy for biomarkers and immune microenvironment: Evidence from RNA-seq. BMC Cardiovasc. Disord..

[24] Nishimura H., Okazaki T., Tanaka Y., Nakatani K., Hara M., Matsumori A., Sasayama S., Mizoguchi A., Hiai H., Minato N. (2001). Autoimmune dilated cardiomyopathy in PD-1 receptor-deficient mice. Science.

[25] Grabie N., Lichtman A.H., Padera R. (2019). T cell checkpoint regulators in the heart. Cardiovasc. Res..

[26] Kamphorst A.O., Wieland A., Nasti T., Yang S., Zhang R., Barber D.L., Konieczny B.T., Daugherty C.Z., Koenig L., Yu K. (2017). Rescue of exhausted CD8 T cells by PD-1-targeted therapies is CD28-dependent. Science.

[27] Garcia-Rivas G., Castillo E.C., Gonzalez-Gil A.M., Maravillas-Montero J.L., Brunck M., Torres-Quintanilla A., Elizondo-Montemayor L., Torre-Amione G. (2020). The role of B cells in heart failure and implications for future immunomodulatory treatment strategies. ESC Heart Fail..

[28] Takeuchi A., Badr M.E.S.G., Miyauchi K., Ishihara C., Onishi R., Guo Z., Sasaki Y., Ike H., Takumi A., Tsuji N.M. (2015). CRTAM determines the CD4+ cytotoxic T lymphocyte lineage. J. Exp. Med..

[29] Koczo A., Marino A., Rocco J., Ewald G., Givertz M.M., Rajagopalan N., Bozkurt B., Elkayam U., Cooper L.T., Fett J. (2021). Proinflammatory TH17 cytokine activation, disease severity and outcomes in peripartum cardiomyopathy. Int. J. Cardiol..

[30] Papa S., Choy P.M., Bubici C. (2019). The ERK and JNK pathways in the regulation of metabolic reprogramming. Oncogene.

[31] Link W., Fernandez-Marcos P.J. (2017). FOXO transcription factors at the interface of metabolism and cancer. Int. J. Cancer.

[32] Masui K., Tanaka K., Akhavan D., Babic I., Gini B., Matsutani T., Iwanami A., Liu F., Villa G.R., Gu Y. (2013). mTOR complex 2 controls glycolytic metabolism in glioblastoma through FoxO acetylation and upregulation of c-Myc. Cell Metab..

[33] Liu L.X., Arany Z. (2014). Maternal cardiac metabolism in pregnancy. Cardiovasc. Res..

[34] Ritterhoff J., Tian R. (2017). Metabolism in cardiomyopathy: Every substrate matters. Cardiovasc. Res..

[35] Davila-Roman V.G., Vedala G., Herrero P., de las Fuentes L., Rogers J.G., Kelly D.P., Gropler R.J. (2002). Altered myocardial fatty acid and glucose metabolism in idiopathic dilated cardiomyopathy. J. Am. Coll. Cardiol..

[36] Hoes M.F., Bomer N., Ricke-Hoch M., de Jong T.V., Arevalo Gomez K.F., Pietzsch S., Hilfiker-Kleiner D., van der Meer P. (2020). Human iPSC-Derived Cardiomyocytes of Peripartum Patients With Cardiomyopathy Reveal Aberrant Regulation of Lipid Metabolism. Circulation.

[37] Schulze P.C., Drosatos K., Goldberg I.J. (2016). Lipid Use and Misuse by the Heart. Circ. Res..

[38] Bankaitis V.A., Garcia-Mata R., Mousley C.J. (2012). Golgi membrane dynamics and lipid metabolism. Curr. Biol..

[39] Lu L., Zhou Q., Chen Z., Chen L. (2018). The significant role of the Golgi apparatus in cardiovascular diseases. J. Cell. Physiol..

[40] Bollen I.A.E., Ehler E., Fleischanderl K., Bouwman F., Kempers L., Ricke-Hoch M., Hilfiker-Kleiner D., Dos Remedios C.G., Kruger M., Vink A. (2017). Myofilament Remodeling and Function Is More Impaired in Peripartum Cardiomyopathy Compared with Dilated Cardiomyopathy and Ischemic Heart Disease. Am. J. Pathol..

[41] Nicin L., Abplanalp W.T., Schanzer A., Sprengel A., John D., Mellentin H., Tombor L., Keuper M., Ullrich E., Klingel K. (2021). Single Nuclei Sequencing Reveals Novel Insights into the Regulation of Cellular Signatures in Children with Dilated Cardiomyopathy. Circulation.

[42] Frangogiannis N.G. (2019). The Extracellular Matrix in Ischemic and Nonischemic Heart Failure. Circ. Res..

[43] Lynch T.L.T., Ismahil M.A., Jegga A.G., Zilliox M.J., Troidl C., Prabhu S.D., Sadayappan S. (2017). Cardiac inflammation in genetic dilated cardiomyopathy caused by MYBPC3 mutation. J. Mol. Cell. Cardiol..

[44] Brodehl A., Belke D.D., Garnett L., Martens K., Abdelfatah N., Rodriguez M., Diao C., Chen Y.X., Gordon P.M., Nygren A. (2017). Transgenic mice overexpressing desmocollin-2 (DSC2) develop cardiomyopathy associated with myocardial inflammation and fibrotic remodeling. PLoS ONE.

[45] Sielemann K., Elbeck Z., Gärtner A., Brodehl A., Stanasiuk C., Fox H., Paluszkiewicz L., Tiesmeier J., Wlost S., Gummert J. (2020). Distinct Myocardial Transcriptomic Profiles of Cardiomyopathies Stratified by the Mutant Genes. Genes.

