# Supplementary material for: Transcriptomic Comparison of Human Peripartum and Dilated Cardiomyopathy Identifies Differences in Key Disease Pathways
Source: J Cardiovasc Dev Dis. 2023 Apr 23;10(5):188. doi: 10.3390/jcdd10050188 (PMC10218903; doi:10.3390/jcdd10050188)
Supplement: Supplementary file 1 [file jcdd-10-00188-s001.zip › Supplementary Figures.pdf]

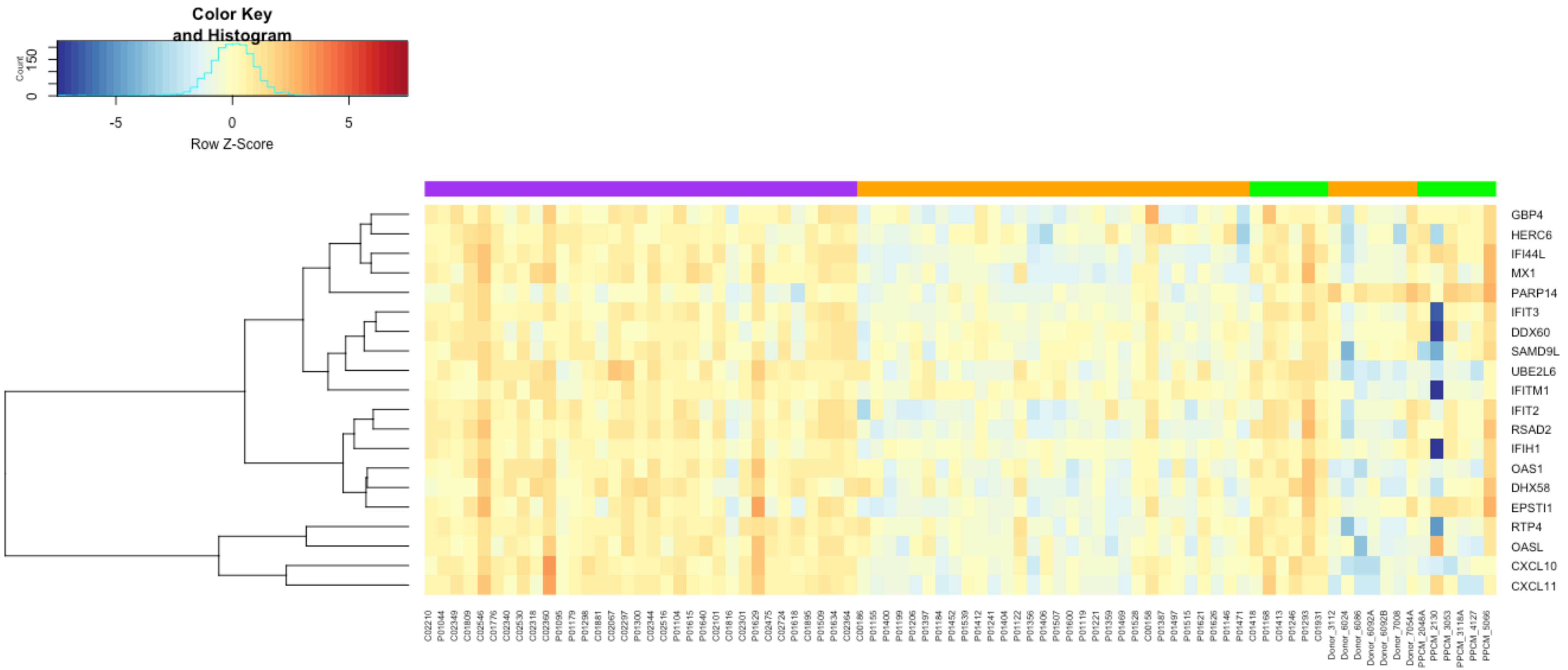

Figure S1. Heatmap of interferon alpha response in PPCM. Purple – DCM, Green – PPCM, Orange – non-failing donors.

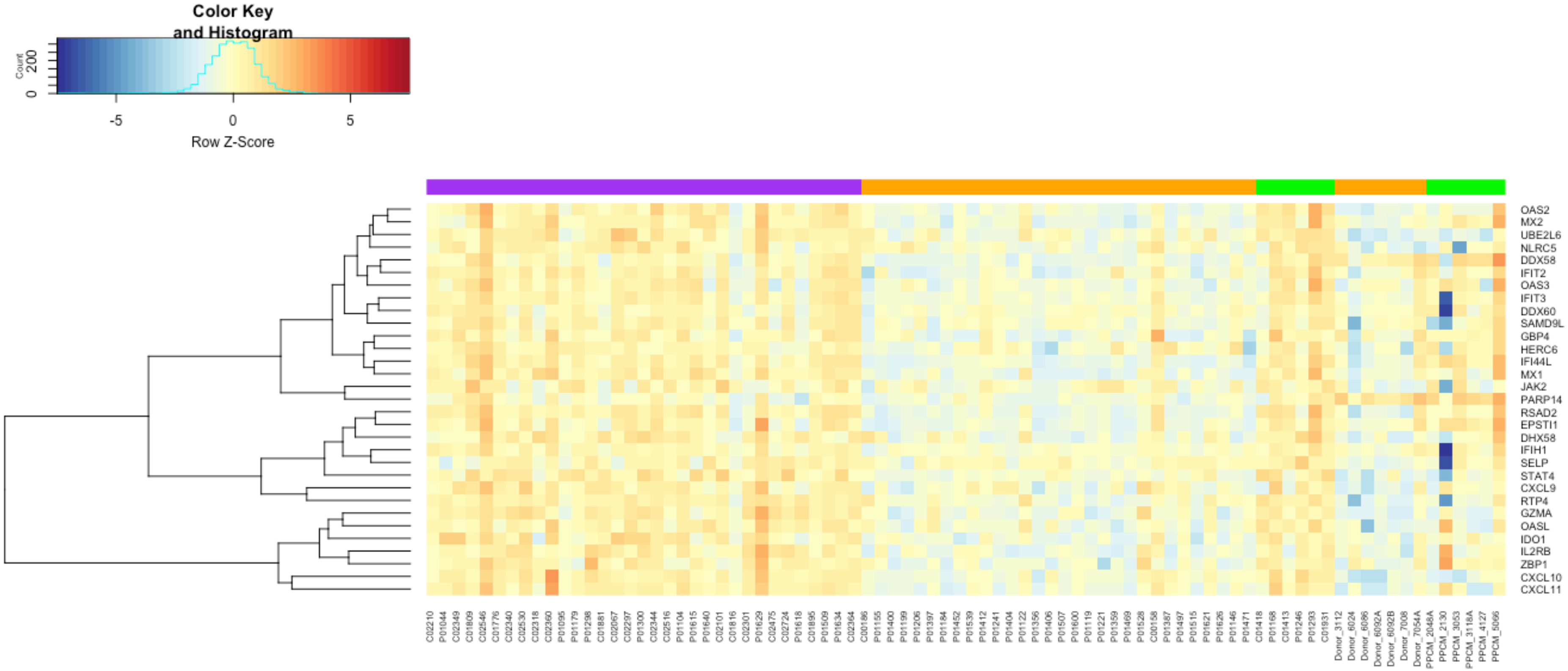

Figure S2. Heatmap of interferon gamma response in PPCM. Purple – DCM, Green – PPCM, Orange – non-failing donors.

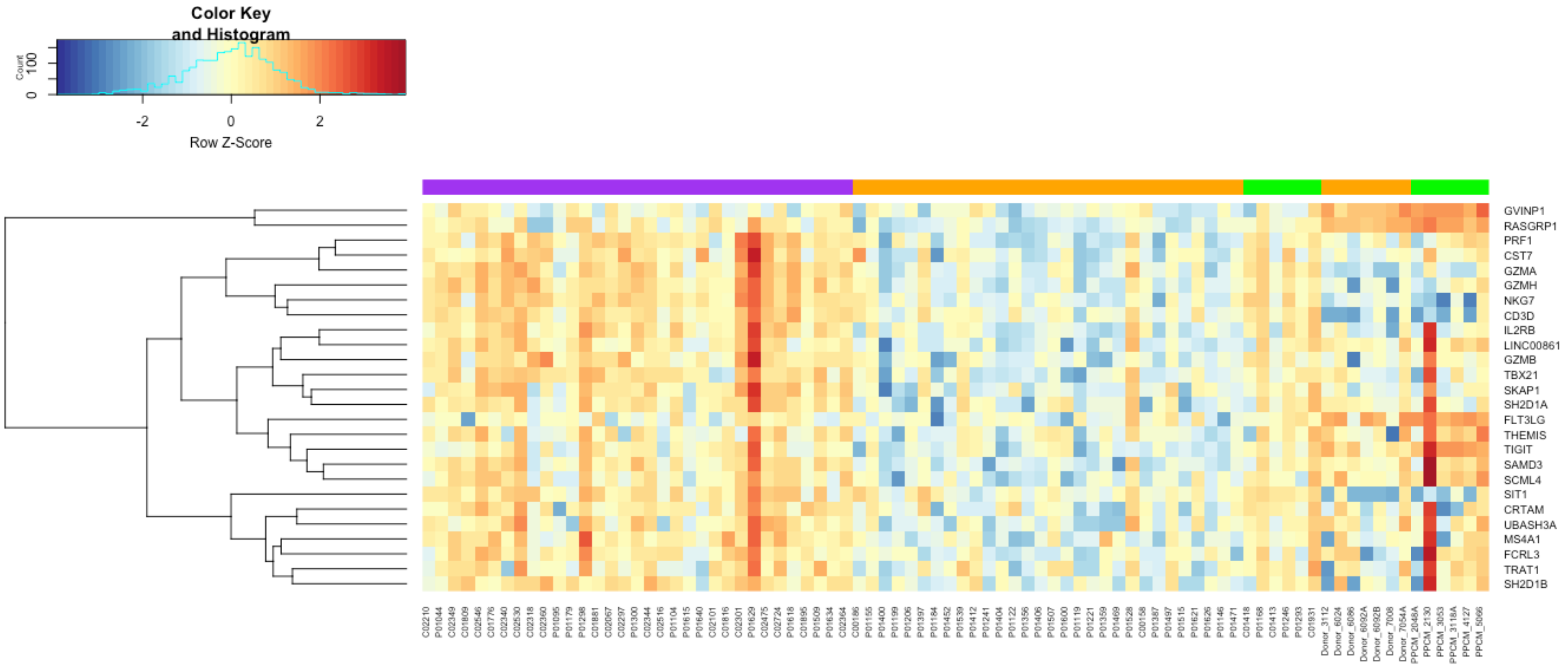

Figure S3. Heatmap of lymphoid cell in heart in PPCM. Purple – DCM, Green – PPCM, Orange – non-failing donors.

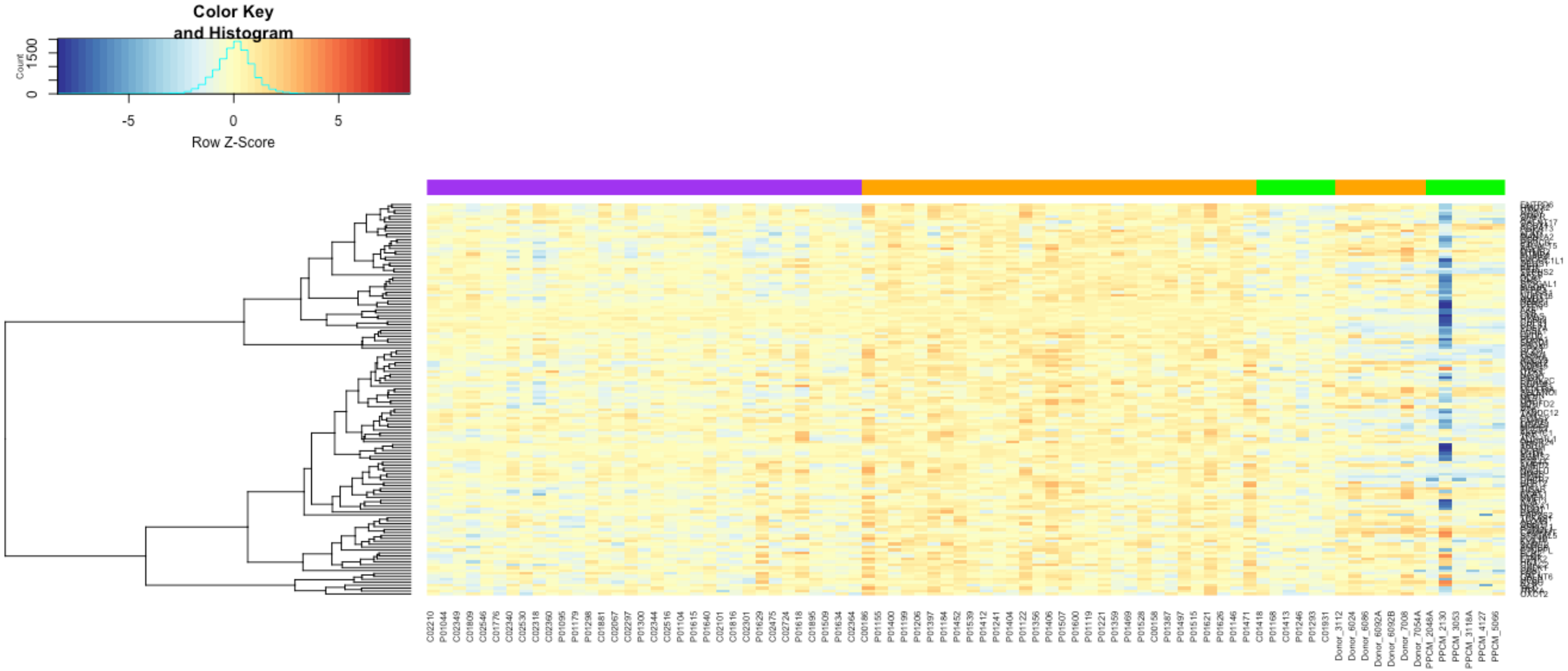

Figure S4. Heatmap of Metabolic pathways in PPCM. Purple – DCM, Green – PPCM, Orange – non-failing donors.

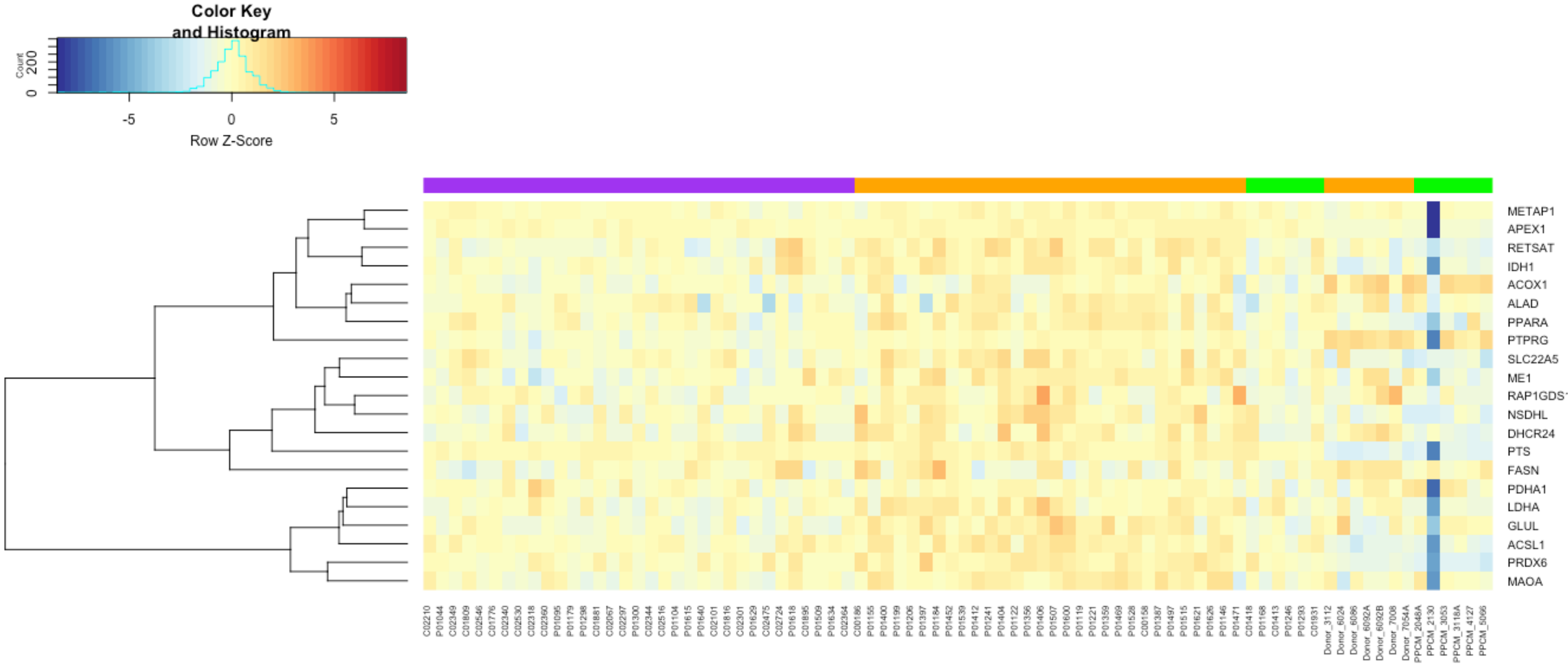

Figure S5. Heatmap of fatty acid metabolism in PPCM. Purple – DCM, Green – PPCM, Orange – non-failing donors.

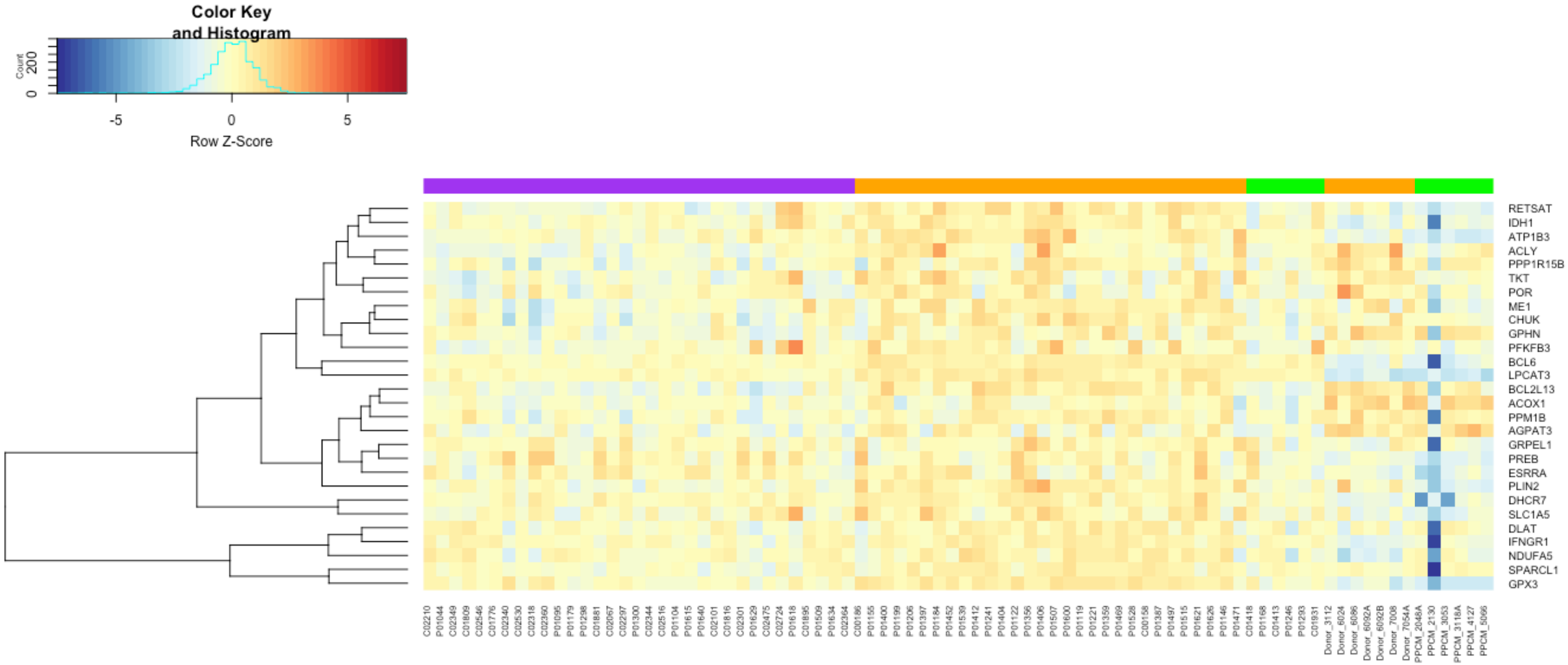

Figure S6. Heatmap of adipogenesis in PPCM. Purple – DCM, Green – PPCM, Orange – non-failing donors.

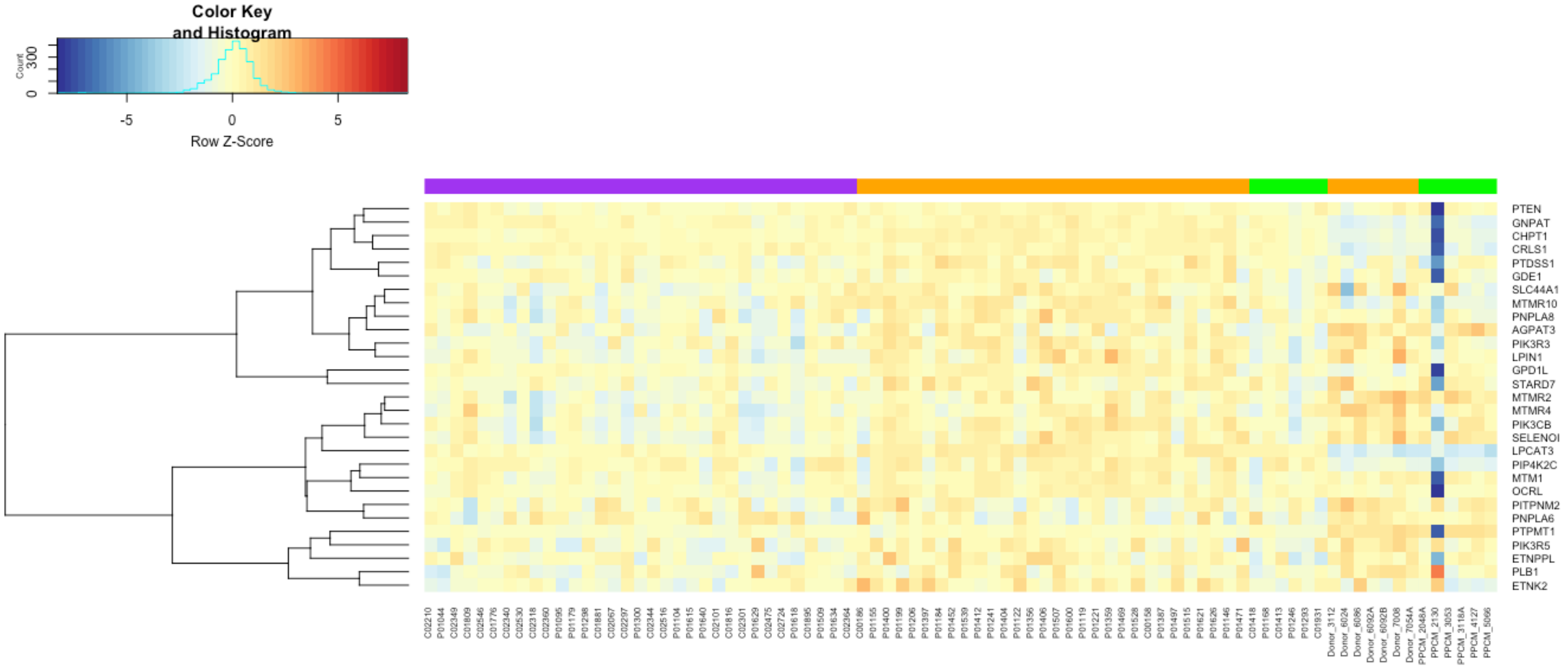

Figure S7. Heatmap of Phospholipid metabolism in PPCM. Purple – DCM, Green – PPCM, Orange – non-failing donors.

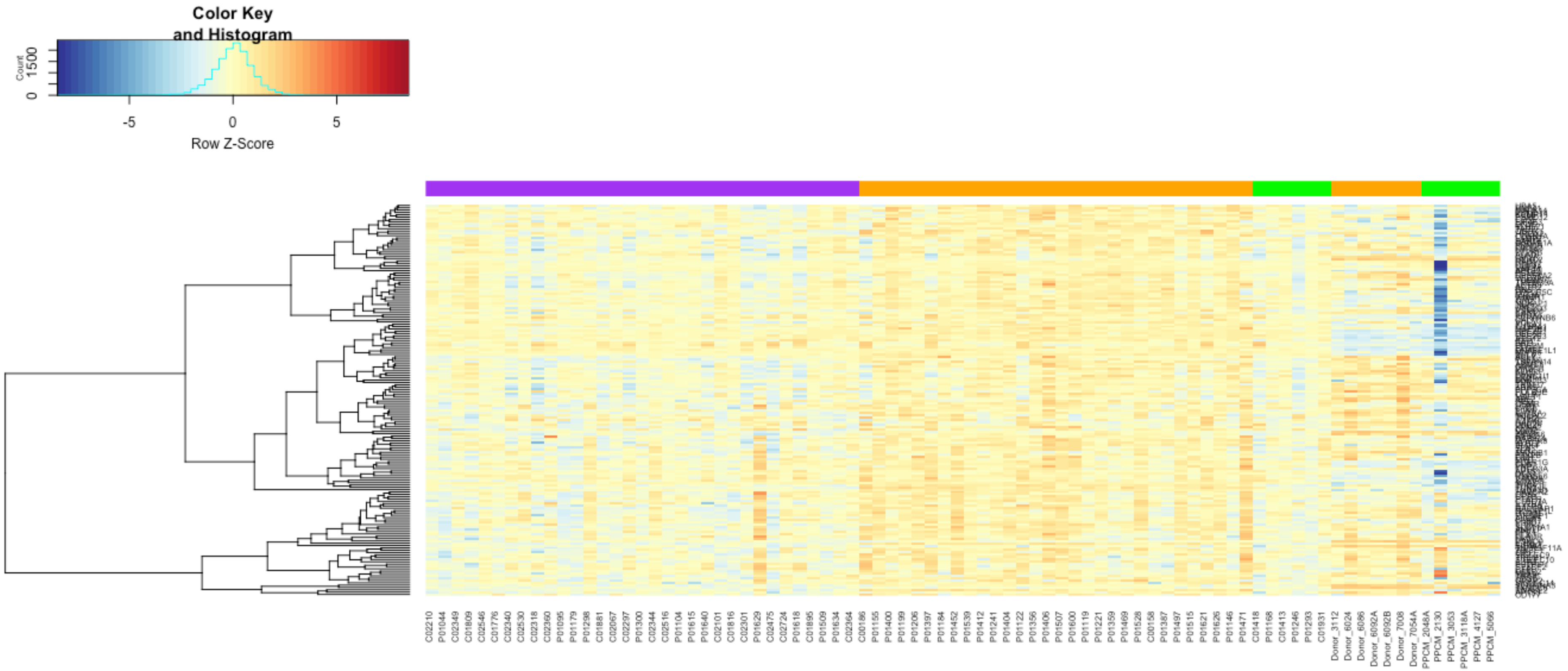

Figure S8. Heatmap of Immune system reactome pathway in PPCM. Purple – DCM, Green – PPCM, Orange – non-failing donors.

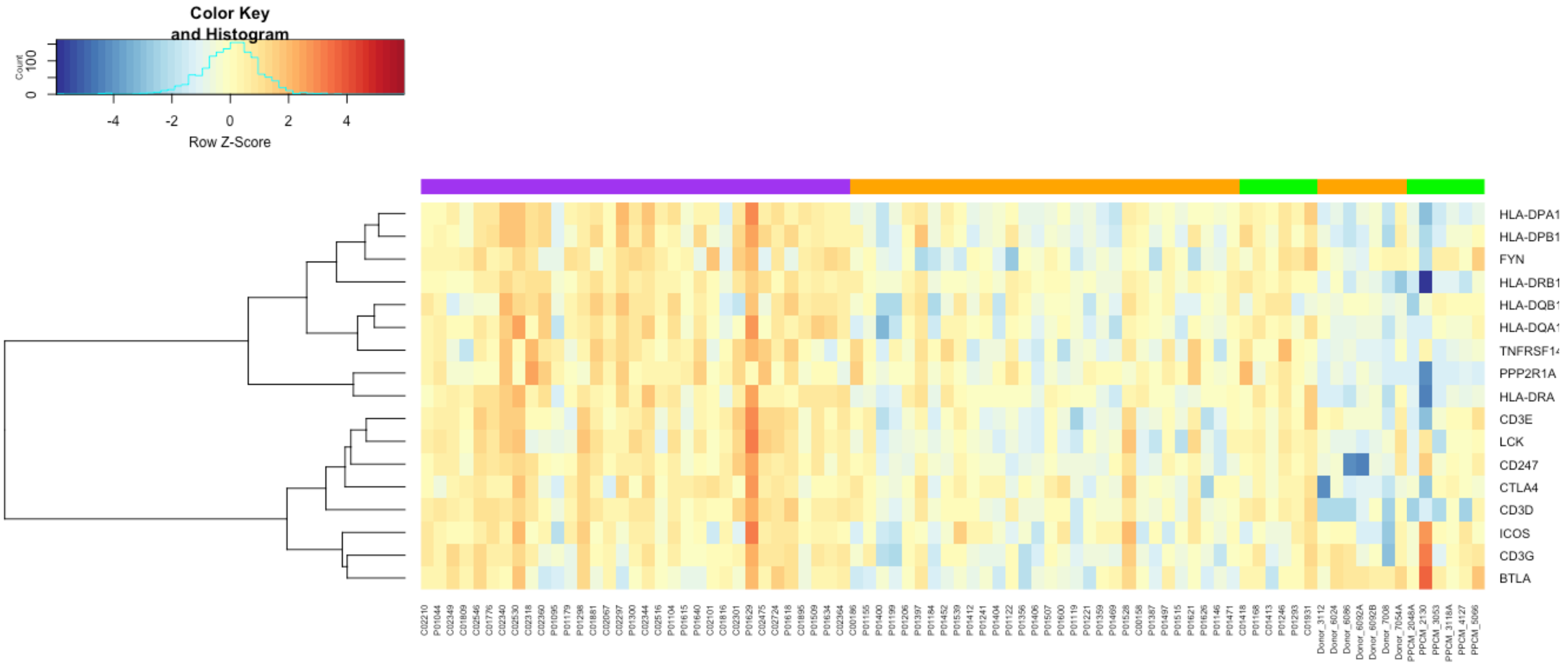

Figure S9. Heatmap of costimulation by CD28 family in DCM. Purple – DCM, Green – PPCM, Orange – non-failing donors.

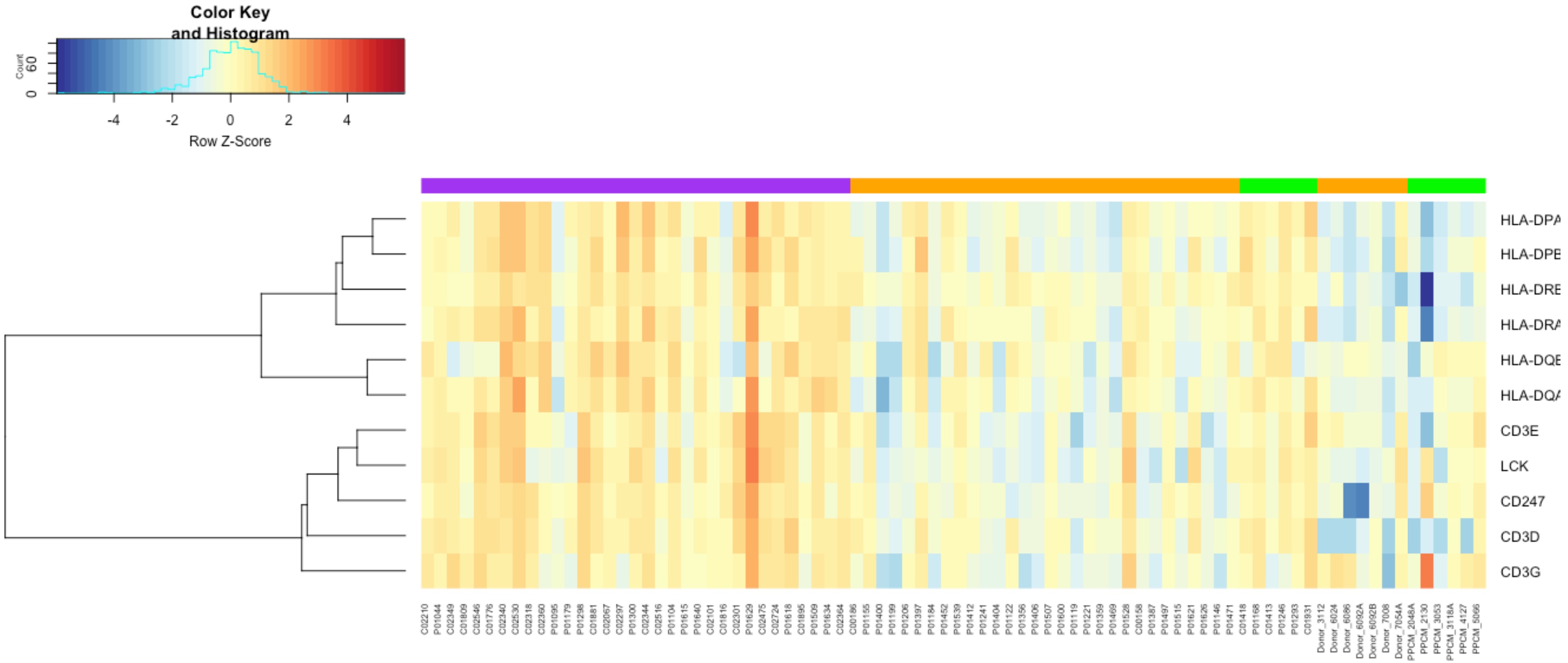

Figure S10. Heatmap of PD1 signaling in DCM. Purple – DCM, Green – PPCM, Orange – non-failing donors.

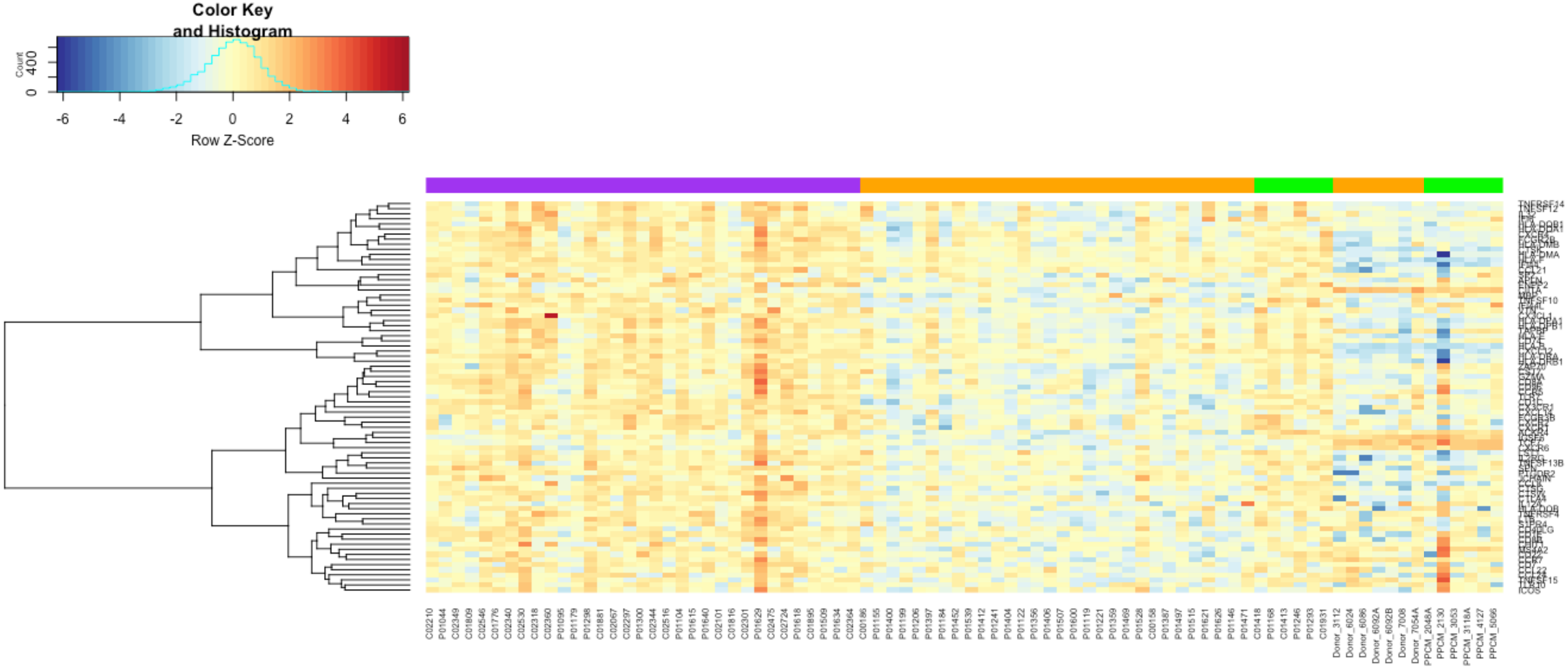

Figure S11. Heatmap of immune response in DCM. Purple – DCM, Green – PPCM, Orange – non-failing donors.

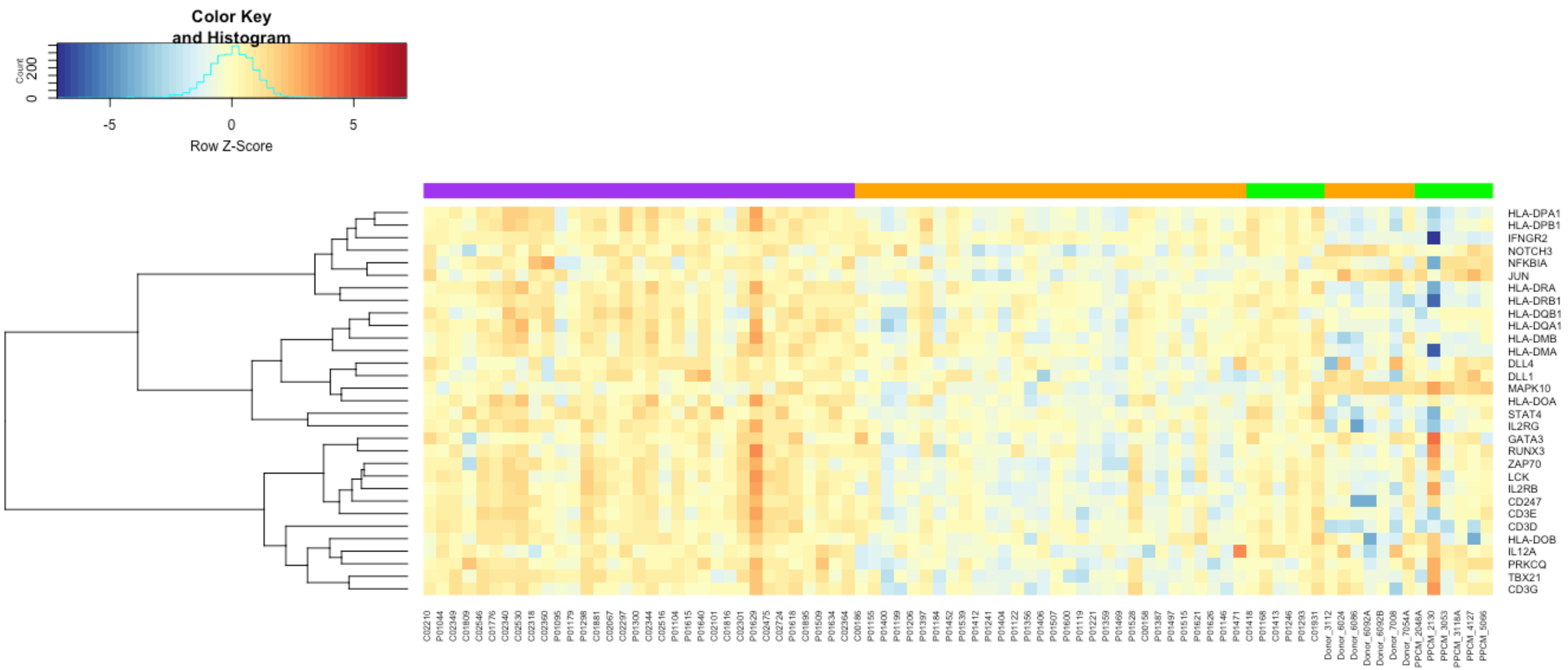

Figure S12. Heatmap of Th1 and Th2 cell differentiation upregulated in DCM. Purple – DCM, Green – PPCM, Orange – non-failing donors.

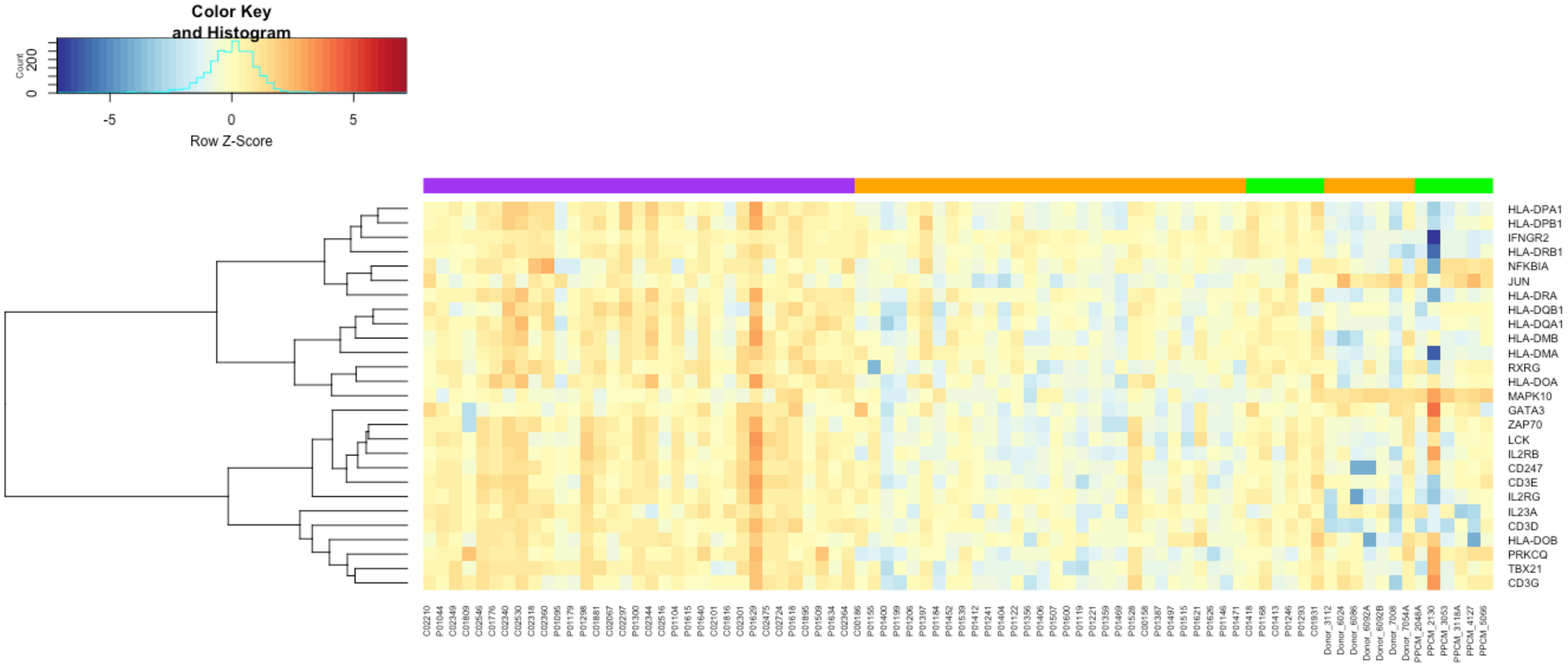

Figure S13. Heatmap of Th17 cell differentiation upregulated in DCM. Purple – DCM, Green – PPCM, Orange – non-failing donors.
